# Determinants of lenalidomide response with or without erythropoiesis-stimulating agents in myelodysplastic syndromes: the HOVON89 trial

**DOI:** 10.1038/s41375-024-02161-6

**Published:** 2024-01-31

**Authors:** A. A. van de Loosdrecht, E. M. P. Cremers, C. Alhan, C. Duetz, F. E. M. in ’t Hout, H. A. Visser-Wisselaar, D. A. Chitu, A. Verbrugge, S. M. Cunha, G. J. Ossenkoppele, J. J. W. M. Janssen, S. K. Klein, E. Vellenga, G. A. Huls, P. Muus, S. M. C. Langemeijer, G. E. de Greef, P. A. W. te Boekhorst, M. H. G. Raaijmakers, M. van Marwijk Kooy, M. C. Legdeur, J. J. Wegman, W. Deenik, O. de Weerdt, T. M. van Maanen-Lamme, P. Jobse, R. J. W. van Kampen, A. Beeker, P. W. Wijermans, B. J. Biemond, B. C. Tanis, J. W. J. van Esser, C. G. Schaar, H. S. Noordzij-Nooteboom, E. M. G. Jacobs, A. O. de Graaf, M. Jongen-Lavrencic, M. J. P. L. Stevens-Kroef, T. M. Westers, J. H. Jansen

**Affiliations:** 1 Department of Hematology, Amsterdam UMC, location VUmc, Cancer Center Amsterdam, Amsterdam, The Netherlands; 2 Department of Laboratory Medicine - Laboratory of Hematology, Radboud University Medical Center, Nijmegen, The Netherlands; 3 Department of Hematology, Radboud University Medical Center, Nijmegen, The Netherlands; 4 HOVON Foundation, Rotterdam, The Netherlands; 5 Department of Hematology, Erasmus MC Cancer Institute, Rotterdam, The Netherlands; 6 Department of Hematology, Meander Medisch Centrum, Amersfoort, The Netherlands; 7 Department of Hematology, University Medical Center Groningen, University of Groningen, Groningen, The Netherlands; 8 Department of Haematology, St. James University Hospital, Leeds, UK; 9 Department of Hematology, Isala Ziekenhuis, Zwolle, The Netherlands; 10 Department of Hematology, Medisch Spectrum Twente, Enschede, The Netherlands; 11 Department of Hematology, Deventer Ziekenhuis, Deventer, The Netherlands; 12 Department of Hematology, Amsterdam UMC, location AMC, Amsterdam, The Netherlands; 13 Department of Internal Medicine, Tergooi Ziekenhuis, Hilversum, The Netherlands; 14 Department of Internal Medicine, St. Antonius Ziekenhuis, Nieuwegein, The Netherlands; 15 Department of Internal Medicine, Dijklander Ziekenhuis, Hoorn, The Netherlands; 16 Department of Internal Medicine, Admiraal de Ruyter Ziekenhuis, Goes, The Netherlands; 17 Department of Internal Medicine, Zuyderland Ziekenhuis, Geleen, The Netherlands; 18 Department of Hematology, Spaarne Gasthuis, Hoofddorp, The Netherlands; 19 Department of Hematology, Haaglanden Ziekenhuis, Den Haag, The Netherlands; 20 Department of Internal Medicine, Groene Hart Ziekenhuis, Gouda, The Netherlands; 21 Department of Internal Medicine, Amphia Ziekenhuis, Breda, The Netherlands; 22 Department of Internal Medicine, Gelre Ziekenhuis, Apeldoorn, The Netherlands; 23 Department of Internal Medicine, Van Weel Bethesda Ziekenhuis, Dirksland, The Netherlands; 24 Department of Internal Medicine, Elkerliek Ziekenhuis, Helmond, The Netherlands; 25 Department of human genetics, Radboud University Medical Center, Nijmegen, The Netherlands; 26 Present Address: Department of Internal Medicine, Rijnstate, Arnhem, the Netherlands; 27 Present Address: Department of General Practice Erasmus MC, Rotterdam, The Netherlands

**Keywords:** Cancer, Haematological cancer

## Abstract

A randomized phase-II study was performed in low/int-1 risk MDS (IPSS) to study efficacy and safety of lenalidomide without (arm A) or with (arm B) ESA/G-CSF. In arm B, patients without erythroid response (HI-E) after 4 cycles received ESA; G-CSF was added if no HI-E was obtained by cycle 9. HI-E served as primary endpoint. Flow cytometry and next-generation sequencing were performed to identify predictors of response. The final evaluation comprised 184 patients; 84% non-del(5q), 16% isolated del(5q); median follow-up: 70.7 months. In arm A and B, 39 and 41% of patients achieved HI-E; median time-to-HI-E: 3.2 months for both arms, median duration of-HI-E: 9.8 months. HI-E was significantly lower in non-del(5q) vs. del(5q): 32% vs. 80%. The same accounted for transfusion independency-at-week 24 (16% vs. 67%), but similar in both arms. Apart from presence of del(5q), high percentages of bone marrow lymphocytes and progenitor B-cells, a low number of mutations, absence of ring sideroblasts, and SF3B1 mutations predicted HI-E. In conclusion, lenalidomide induced HI-E in patients with non-del(5q) and del(5q) MDS without additional effect of ESA/G-CSF. The identified predictors of response may guide application of lenalidomide in lower-risk MDS in the era of precision medicine. (EudraCT 2008-002195-10).

## Introduction

Myelodysplastic syndromes (MDS) are characterized by peripheral blood cytopenia, dysplasia of bone marrow (BM) cells and propensity to progress towards acute myeloid leukemia. Erythropoiesis-stimulating agents (ESA) are considered as first-line treatment in case of low erythropoietin levels and no or limited transfusion dependency [1–4]. Erythroid responses may increase when ESA are combined with granulocyte-colony stimulating factor (G-CSF) [2, 4]. Lenalidomide is one of the second-line treatment modalities in lower-risk MDS, in particular in MDS with 5q deletion (del(5q)). High sensitivity of MDS with del(5q) to lenalidomide is evidenced by erythroid hematologic improvement (HI-E) in 60–70% and cytogenetic remission in more than 50% of patients [5]. In lower-risk non-del(5q) MDS, lenalidomide resulted in approximately 25–35% HI-E and 20% red blood cell (RBC) transfusion independency (TI) [6–8]. Combination with ESA showed additive effects on HI-E (up to 40%) and TI (up to 25%) [7, 8].

Lenalidomide binds to the ubiquitin-E3 ligase cereblon, altering its substrate specificity and leading to drug-induced CK1a and IKFZ1/4 degradation. This is thought to be of therapeutic importance in MDS and multiple myeloma, respectively. Del(5q) MDS cells are particularly sensitive to lenalidomide as the CK1a encoding gene is located on the long arm of chromosome 5. In del(5q) cells, haploinsufficiency leads to low expression of CK1a, further decreased by lenalidomide, inducing TP53-dependent apoptosis [9, 10]. Lenalidomide was reported to stabilize the erythropoietin receptor by inhibiting E3 ubiquitin ligase RNF41, thereby promoting accumulation of signaling complexes that restore response to erythropoietin [11, 12]. Besides, lenalidomide has shown broad immunomodulatory activities towards enhancement of anti-tumor properties of innate and adaptive immunity [13]. Although studies identified molecular predictors of response to lenalidomide in non-del(5q) MDS, predictors of response still need to be better defined [14, 15]. Herein, we report the final analysis of the HOVON89 phase-II randomized multicenter clinical trial in low and intermediate-1 risk MDS investigating effects of lenalidomide with HI-E as primary endpoint. ESA and G-CSF were added only if HI-E was not attained. Additionally, we studied potential predictors of response using extensive profiling by flow cytometry (FC) and next-generation sequencing (NGS).

## Patients and methods

### Inclusion and exclusion criteria

Eligible patients were aged ≥ 18 years with low or int-1 risk (IPSS ≤ 1) MDS (non-del(5q) and del(5q)) or CMML-1 (WBC ≤ 12 × 10^9^/L) according to WHO2001 [16]. Patients with no response to first-line ESA/G-CSF or relapsed after hematologic improvement (HI) were included (inclusion and exclusion criteria see protocol (Table 1, supplementary text)). Patients with a low probability of response to standard ESA/G-CSF (serum EPO ≥ 200U/l and ≥2 units RBC/month for at least 8 weeks; units must be given for a Hb ≤ 5.6 mmol/L (9 g/dl)) were also included. The study was approved and registered at www.trialregister.nl; NTR1825 (former ID); NL1715 (recent ID); EudraCT 2008-002195-10; METC: 2009/50 NL25632.029.08. Accrual of patients started May 27, 2009; the target number of 200 patients was reached on August 12, 2015. All patients signed informed consent.

**Table 1:** Baseline characteristics of patients in the HOVON89 study.

	Arm A	Arm B	Total
*n*	92	92	184
*Age (y)*,			
median (range)	71 (41–84)	71 (38–89)	71 (38–89)
*Sex, n (%)*			
Male	51 (55)	49 (53)	100 (54)
Female	41 (45)	43 (47)	84 (46)
*WHO performance, n (%)*			
WHO 0	36 (39)	35 (38)	71 (39)
WHO 1	50 (54)	48 (52)	98 (53)
WHO 2	5 (5)	5 (5)	10 (5)
unknown	1 (1)	4 (4)	5 (3) (*p* = 0.683)
*WHO classification, n (%)*			
RA	2 (2)	-	2 (1)
RARS	13 (14)	15 (16)	28 (15)
RCMD	21 (23)	21 (23)	42 (23)
RCMD-RS	28 (30)	19 (21)	47 (26)
RAEB-1	9 (10)	14 (15)	23 (13)
MDS-U	3 (3)	1 (1)	4 (2)
CMML-1	3 (3)	4 (4)	7 (4)
Del (5q)	13 (14)	17 (18)	30 (16)
unknown	-	1 (1)	1 (1) (*p* = 0.605)
*IPSS, n (%)*			
0	42 (46)	35 (38)	77 (42)
0.5	38 (41)	39 (42)	77 (42)
1	12 (13)	17 (18)	29 (16)
1.5	-	1 (1)^a^	1 (1)^a^ (*p* = 0.486)
*Baseline, median (range)*			
Hb in: (mmol/l)	5.3 (3.6–7.6)	5.5 (3.7–8.1)	5.4 (3.6–8.1)
Hb in: (g/dl)	8.5 (5.8–12.3)	8.9 (6.0–13.1)	8.7 (5.8–13.1)
Platelets (0.10^9^/l)	224 (31–964)	199 (33–636)	215 (31–964)
WBC (0.10^9^/l)	4.4 (1.1–14.0)	4.1 (1.5–15.7)	4.3 (1.1–15.7) (*p* = 0.271)
*Baseline Epo, median U/l (range)*	248 (15–968)	192 (7–859)	206 (7–968) (*p* = 0.129)
*Pretreatment ESA/G-CSF* *n*, (%)^b^	66 (72)	57 (62)	123 (67) (*p* = 0.394)

^a^indicates a patient with IPSS score 1.5: this patient was included in the study based on an initial diagnosis of RCMD with int-1 risk; renewed BM analysis > 3 months revealed additional chromosomal abnormalities which could be confirmed in the samples of BM at entry of the study. Patient continued in the study, but was upgraded to IPSS 1.5.

^b^Baseline median EPO level in patients exposed to ESA/G-CSF was: 127 U/l (range 7–781). Twenty-one patients stopped ESA/G-CSF within 1 month before study. The median EPO level of these patients was: 104 U/l (19–690). The median EPO level of patients who were not previously exposed to ESA/G-CSF was significantly higher: 589 U/l (*p* = 0.000; range 24–968) without differences between arms A and B.

### Study design

Randomization was performed at inclusion between lenalidomide (Revlimid^TM^) (arm A) or combined with (arm B) step-wise dosed ESA (NeoRecormon™) and G-CSF (Neupogen™), guided by hematologic improvement (HI). Patients were treated for a minimum of 6 months (arm A) and 12 months (arm B) or until disease progression (Fig. S1, supplementary text).

### Study objectives and endpoints

The primary objective was to evaluate the efficacy of lenalidomide with or without ESA/G-CSF in terms of erythroid response (HI-E) and HI defined by IWG2006 [17]. Secondary objectives were safety and tolerability, time-to-HI(-E), duration of-HI(-E), progression-free survival (PFS), overall survival (OS), and RBC transfusion requirements (supplementary text). The primary endpoint was HI-E and HI according to IWG2006 [17]. Final analysis was performed six years after the last patient entered the study (June 24, 2021).

### Baseline biomarker studies by flow cytometry and next-generation sequencing

Baseline BM aspirates were processed for FC and NGS to explore biomarkers for response. FC analyses were conducted according to ELNet guidelines [18]. (supplementary text, Table S1). For NGS, DNA was isolated from BM mononuclear cells using nucleospin columns (BioKe, The Netherlands). Sequencing was performed using IonTorrent PGM and Illumina NextSeq technology [19] (Table S2, supplementary text). NGS data-processing and variant-calling is described in the supplementary text.

### Statistical methods

All analyses were performed according to the intention-to-treat principle. Statistical methods are described in supplementary text. Difference in response rate in terms of HI and HI-E between the two arms was computed together with 80% CI. All point estimates of the secondary survival endpoints are accompanied by 95% CI. Kaplan–Meier survival curves and Cox regression tests were used to compare survival distributions between treatment arms.

## Results

### Patients

**Fig. 1:**
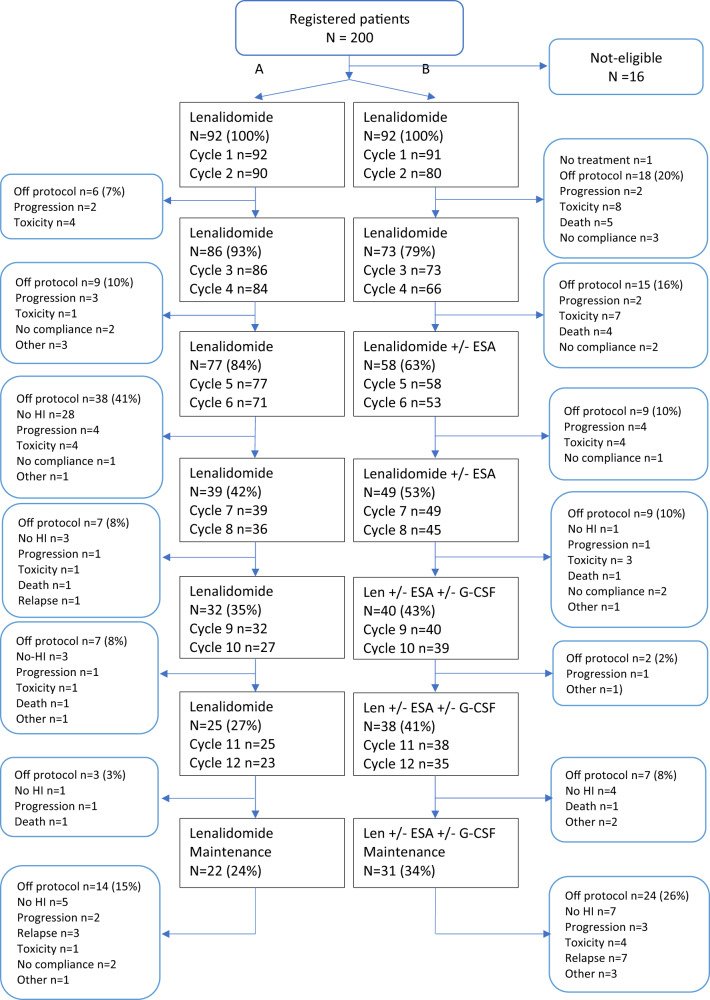
CONSORT diagram. Two hundred patients were registered. Sixteen patients were ineligible. Seven patients appeared to be higher risk IPSS 1.5 and two patients were not treated with standard ESA/G-CSF before study entry. One patient appeared to be not lenalidomide-naïve. In three patients the diagnosis of MDS could not be confirmed, one patient suffered from a second active malignancy, one patient had ANC counts < 0.8 at study entry and one patient was registered twice.

A total number of 200 MDS patients were randomly assigned to lenalidomide (arm A) or lenalidomide with ESA/G-CSF (arm B). The final analysis included 184 patients, 92 in each arm (Supplementary text, Fig. 1, Table 1); median follow-up was 70.7 months (95% CI: 61.9–92.5). Baseline characteristics were comparable among treatment arms (Table 1). Median time from diagnosis of MDS to study entry was 19.0 months (range: 0.3–251). Thirty percent of the patients did not receive ESA/G-CSF before study entry, 67% received ESA/G-CSF and 3% other treatments (3 ciclosporine, 1 danazol, 2 pyridoxine). The median number of RBC units transfused was 13 (range 0–72) with a median number of 4 (range 0–13) within 4 weeks prior to study. None of the patients received platelet transfusions before study entry.

### Treatment characteristics

Ninety-three percent of patients in arm A and 79% of patients in arm B received lenalidomide either as full dose, full dose with delay or with >10% dose reduction and/or delay in the first 4 cycles (see also CONSORT diagram Fig. 1). A relatively high percentage of patients went off-protocol after 4 cycles due to progression, toxicity, death or no compliance (arm A 16%, arm B 36%, *p* = 0.003). After 4 cycles, no differences in HI-E were observed between arms (arm A 38%, arm B 39%). In arm B, ESA was added to lenalidomide in only those patients who did not achieve HI-E after 4 cycles of lenalidomide; in these non-responders, a cumulative percentage of 94% of patients were exposed to ESA in cycles 5–12. Patients who did not achieve HI-E after exposure to lenalidomide and ESA, received G-CSF. In cycles 9–12, a cumulative percentage of 56% of patients received G-CSF.

### Erythroid response

Forty percent of patients achieved HI-E (arm A 39%, arm B 41%; *p* = 0.764; Table 2). Median time-to-HI-E was 3.2 months (arm A: 3.1, arm B 3.5). HI-E at week 24 was 27% (arm A 24%, arm B 29%). The median duration of HI-E was 9.8 months (arm A 11.2, arm B 9.3). Sixty-eight patients (37%) became transfusion independent (TI) after median 3.0 months without differences between arms. Forty-four out of 49 (90%) patients achieving HI-E were TI at week 24 (including three patients who were TI before start of treatment), which is 24% of the total cohort (arm A 23%, arm B 25%).

**Table 2:** Responses to lenalidomide with or without ESA/G-CSF according to IWG2006; arm A vs. arm B.

	Arm A	Arm B	Total
*n*	92	92	184
HI-E, *n* (%)	36 (39%)	38 (41%)	74 (40%)
HI (HI-E/P/N), *n* (%)	32 (35%)	33 (36%)	65 (35%)
Time-to-HI-E, median months (range) Time-to-HI-E, median weeks (range)	3.1 (1.8–8.2) 12.2 (7.2–33)	3.5 (1.6–12.3) 14.1 (6.4–49.3)	3.2 (1.6–12.3) 12.6 (6.4–49.3)
Time-to-HI (HI-E/P/N), median months (range)	3.2 (1.8–8.2)	3.6 (1.6–11)	3.5 (1.6–11)
Duration of HI-E, median months (range)	11.2 (1.5–135)	9.3 (0.7–107)	9.8 (0.7–135)
Duration of HI, median months (range)	11.2 (1.5–135)	9.4 (0.7–107)	10.1 (0.7–135)
HI-E at 24 weeks, *n* (%) HI at 24 weeks, *n* (%)	22 (24%) 19 (21%)	27 (29%) 25 (27%)	49 (27%) 44 (24%)
TI, *n* (%) Time-to-TI, median months (range) Duration of TI, median months (range) TI at 24 weeks, *n* (%)	35 (38%) 3.0 (1.8–8.2) 11.4 (1.5–135) 21 (23%)	33 (36%) 2.9 (1.6–12.3) 10.4 (0.7–107) 23 (25%)	68 (37%) 3.0 (1.6–12.3) 11.1 (0.7–135) 44 (24%)
CR, CRi, CRd, incl. TI, *n* (%) SD^a^, *n* (%) no response, *n* (%) PD, *n* (%)	30 (32%) 38 (41%) 13 (14%) 11 (12%)	27 (29%) 30 (33%) 28 (30%) 7 (8%)	57 (31%) 68 (37%) 41 (22%) 18 (10%)

No significant differences between arms A and B in all comparisons.

*CR* complete remission, *CRi* complete remission with incomplete peripheral blood recovery, *CRd* complete remission with persistence of dysplasia, *HI* hematologic improvement, *HI-E/P/N* hematologic improvement- erythroid/platelet/neutrophil, *TI* transfusion independency, *MDS* myelodysplastic syndromes, *RS* ring sideroblasts, *n* number, *PD* progressive disease, *SD* stable disease.

^a^1 patient with RAEB-1 (IPSS 1) with 8% BM blasts at study entry met the criteria of marrow CR assigned to SD.

### Platelet response, neutrophil response, and HI

We could only evaluate platelet and neutrophil responses in patients with pretreatment thrombocytopenia (*n* = 49) and neutropenia (*n* = 33). Few patients achieved a platelet and/or neutrophil response, (5/49 (10%) and 2/33 (6%), respectively). HI including HI-E was 35%, and similar between arms (arm A 35%, arm B 36%). The time-to-HI was 3.5 months (arm A 3.2, arm B 3.6) with a median duration of HI of 10.1 months (arm A 11.2, arm B 9.4). The median duration of HI of 10 patients without loss of HI until date of last contact was 91.8 months (range 65–135) and did not differ between arms (Table 2).

### Complete remission and cytogenetic response

We assessed complete remission (CR), CR with incomplete peripheral blood recovery (CRi) and CR with persistence of dysplasia (CRd) according to IWG2006 criteria [17]. Thirty-one percent achieved CR, CRi, or CRd (Table 2). Twenty-one patients fulfill the criteria of CR (11%) of whom 18 presented with <5% blasts at study entry. Cytogenetic response (including FISH) was evaluable in 116/184 patients (63%); almost half of these (*n* = 61; 33% of total cohort) showed a normal karyotype at baseline. In 68 patients (37%), follow-up cytogenetics was not available. Cytogenetic response was complete in 13% and partial in 5% of cases without differences between arms; 10% achieved no response. We observed loss of cytogenetic response in one case and additional aberrations in two cases.

### Progression-free survival (PFS) and overall survival (OS)

**Fig. 2:**
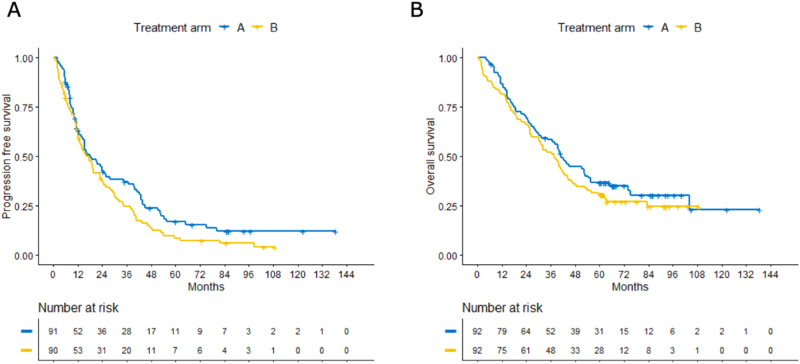
Progression-free survival and overall survival. Kaplan–Meier-estimated progression-free survival (PFS, panel **A**) and overall survival (OS, panel **B**) by arm A vs. arm B (HOVON89).

Median PFS in all patients was 17.4 months (CI 13.6–23.0); arm A 18.1 (CI 12.7–25.7) and arm B 16.1 (CI 12.1–23.1) (Fig. 2A); median OS was 39.9 months (CI 31.4–44.2); arm A 41.0 (CI 30.2–54.2) and arm B 37.7 (CI 26.7–44.2) (Fig. 2B). Median survival of patients still alive was 67.1 months (range 6.5–139) without differences between arms (arm A 66.8, range 6.5–139; arm B 67.8, range 60.2–139).

### Leukemic evolution

Leukemic evolution was determined with competing risk of death without leukemic transformation (Fig. S2). Leukemic evolution occurred in 31 patients (17%) (arm A *n* = 16 (17%), arm B *n* = 15 (16%)); median time-to leukemic evolution was 11 months (range 2–44) and similar between IPSS risk groups (not shown).

### MDS with isolated del(5q) versus non-del(5q)

MDS with isolated del(5q) is highly sensitive to lenalidomide [5]. This subgroup covered 16% (*n* = 30) of included patients (arm A, *n* = 13 (14%), arm B, *n* = 17 (18%), *p* = 0.426). Eighty percent of the del(5q) patients achieved HI-E, 11 in arm A, 13 in arm B with median time-to-response of 2.8 months (arm A 2.9, arm B 2.8; Table 3). Median duration of HI-E was 11.5 months (arm A 15.4, arm B 10.4). Thirty-two percent of 154 non-del(5q) patients achieved HI-E (32% arm A, 33% arm B (*p* = 0.824)), with median time-to-HI-E of 3.4 months and median duration of response of 8.3 months. TI at week 24 was 67% for del(5q) vs. 16% for non-del(5q) (*p* < 0.001) but not different between arms. Median duration of TI was 13.7 (range 3–101) and 9.3 (range 0.7–135) months for del(5q) and non-MDS del(5q), respectively. This difference was not statistically different, possibly due to the low number of patients. Despite significant differences in HI-E and TI at week 24 between del(5q) and non-del(5q), we observed no significant differences in PFS (*p* = 0.81) and OS (*p* = 0.61) (Fig . S4a, b). Results did not change after reclassifying patients with chromosome 5 abnormalities according to WHO2016 definitions [20] (see supplementary text).

**Table 3:** Responses according to IWG2006: MDS non-del(5q) vs. MDS del(5q).

	Non-del(5q)	Del(5q)	Total cohort
*n*	154	30	184
HI-E, *n* (%)	50 (32%)	24 (80%)	74 (40%) (*p* < 0.001)
HI (HI-E/P/N), *n* (%)	43 (28%)	22 (73%)	65 (35%) (*p* < 0.001)
Time-to-HI-E, median months (range) Time-to-HI-E, median weeks (range)	3.4 (1.8–12.3) 13.6 (7.2–49.3)	2.8 (1.6–4.6) 11.3 (6.4–18.4)	3.2 (1.6–12.3) 12.6 (6.4–49.3)
Time-to-HI (HI-E/P/N), median months (range)	3.6 (1.8–11.0)	3.0 (1.6–4.8)	3.5 (1.6–11.0)
Duration of HI-E, median months (range)	8.3 (0.7–135)	11.5 (2.7–101)	9.8 (0.7–135)
Duration of HI, median months (range)	9.2 (0.7–135)	11.5 (2.7–101)	10.1 (0.7–135)
HI-E at 24 weeks, *n* (%) HI at 24 weeks, *n* (%)	29 (19%) 27 (18%)	20 (67%) 17 (57%)	49 (27%) (*p* < 0.001) 44 (24%) (*p* < 0.001)
TI, *n* (%) Time-to-TI, median months (range) Duration of TI, Median months (range) TI at 24 weeks, *n* (%)	46 (30%) 3.2 (1.8–12.3) 9.3 (0.7–135) 24 (16%)	22 (73%) 2.8 (1.6–4.6) 13.7 (3.0–101) 20 (67%)	68 (37%) (*p* < 0.001) 3.0 (1.6–12.3) 11.1 (0.7–135) 44 (24%) (*p* < 0.001)
CR, CRi, CRd incl. TI, *n* (%) SD, *n* (%) no response, *n* (%) PD, *n* (%)	37 (24%) 63 (41%) 36 (23%) 18 (12%)	20 (66%) 5 (17%) 5 (17%) 0 (0%)	57 (31%) 68 (37%) 41 (22%) 18 (10%)

*p*-values represent significance levels between MDS non-del(5q) vs. MDS del(5q). Due to the low number of CMML patients (*n* = 7), we did not report separately on hematological response in this subgroup.

*CR* complete remission, *CRi* complete remission with incomplete peripheral blood recovery, *CRd* complete remission with persistence of dysplasia, *HI* hematologic improvement, *HI-E/P/N* hematologic improvement- erythroid/platelet/neutrophil, *TI* transfusion independency, *MDS* myelodysplastic syndromes, *RS* ring sideroblasts, *n* number, *PD* progressive disease, *SD* stable disease.

### Survival by HI-E via landmark analysis at 12 months

We performed a landmark analysis at 12 months to evaluate the influence of reaching HI-E on OS. Median OS was significantly higher in responders (HI-E) (71 months; CI 36.4-not reached) vs. non-responders (28 months; CI 18.8–31.1; *p* < 0.001) (Fig. S5a). Stratified for MDS del(5q) and non-del(5q), OS was significantly longer for responders (HI-E) vs. non-responders (*p* = 0.008 and *p* < 0.001, respectively) (Fig. S5b, c).

### Adverse events

A relatively high number of adverse events (AE) of grade 2, 3, and 4 was reported. Within the first 12 cycles, the rate of any AE of grade 2, 3, and 4 was 90% (arm A) and 95% (arm B); grade 3 and 4 AE concerned 65% (arm A *vs*. 72% arm B) (n.s.; Table S3a). For any toxicity grade 2, 3, and 4, most AE were reported within the first 4 cycles, arm A 81% and arm B 88%; any toxicity grade 3 and 4 concerned 51% (arm A) vs. 56% (arm B)(n.s.; Table S3b). Most of the reported grade 2, 3, and 4 AE concerned hematological toxicity. Grade 2 toxicities were mainly constitutional symptoms, gastrointestinal complaints, and skin abnormalities.

### Determinants of response to lenalidomide with or without ESA/G-CSF

IPSS subgroup, time from diagnosis to treatment, previous treatment with ESA/G-CSF, baseline EPO level and pretreatment transfusion load did not predict HI-E (Fig. S3a–c, supplementary text, data not shown). None of these parameters were related to PFS or OS in univariate analysis (data not shown). Absence of ring sideroblasts in non-del(5q) significantly predicted for reaching HI-E (*p* = 0.029) and TI (*p* = 0.037); (Table S4). Ring sideroblasts were associated with presence of a SF3B1 mutation in 84% and 86% of MDS-RARS and RCMD-RS, respectively.

### Flow cytometry

Adequate samples for FC analysis were available in 129 of 141(91%) patients with informed consent. We investigated three FC scores, the integrated flow score (iFS), the Ogata score, and the flow cytometric scoring system (FCSS) (see supplementary text) [18, 21–23]. Percentages of several BM cell populations appeared predictive for HI-E, duration of-HI-E, OS, and PFS, whereas the iFS, Ogata score, and FCSS were solely predictive for PFS and OS (Table S5). Low percentages of BM neutrophils and high percentages of BM lymphocytes were favorably associated with HI-E in the total cohort and when stratified for del(5q). Progenitor B-cell percentages were favorably associated with HI-E in the total cohort as well as in non-del(5q); myeloid progenitor percentages were negatively associated with HI-E in non-del(5q). Higher progenitor B-cell and lower myeloid progenitor percentages were predictive for prolonged OS and PFS in all patients and non-del(5q).

**Fig. 3:**
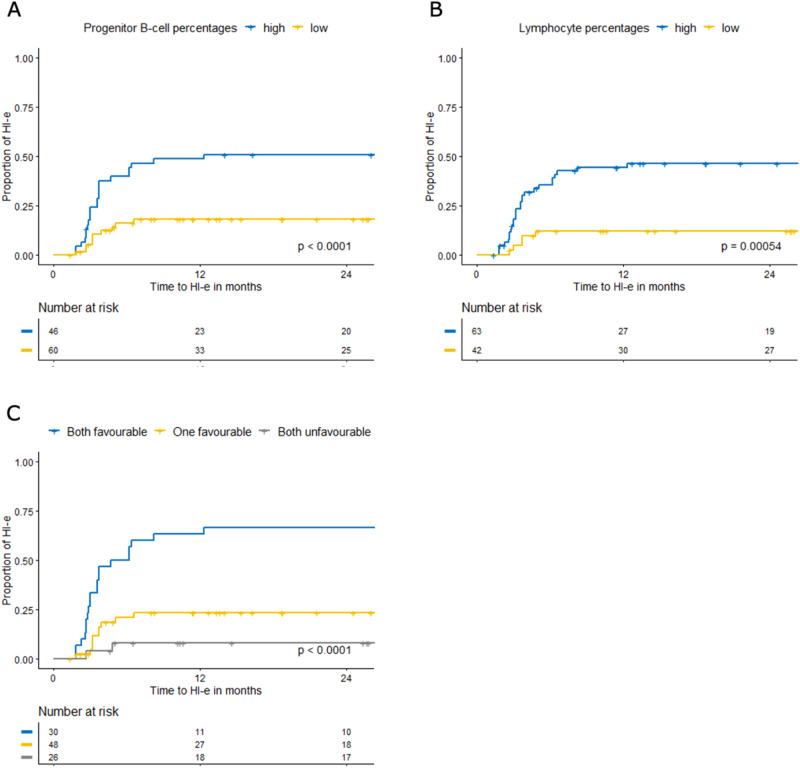
Determinants of response to lenalidomide by flow cytometry in non-del(5q) MDS. Kaplan–Meier-estimated erythroid hematological Improvement (HI-E) according to IWG2006; **A** percentages of BM progenitor B-cells; **B** percentages of bone marrow (BM) total lymphocytes; **C** combination of percentage of BM lymphocytes and progenitor B-cells.

When combining all FC parameters in a multivariate analysis, only BM lymphocyte percentages independently predicted HI-E in all MDS (HR 1.064, *p* < 0.001) and del(5q) (HR 1.135, *p* < 0.001; Table S5). In non-del(5q), both lymphocyte (HR 1.04, *p* = 0.02) and progenitor B-cell (HR 1.023, *p* = 0.04) percentages proved independently predictive. Given the need to identify non-del(5q) patients that may respond to lenalidomide, we determined the best cut-off based on optimization of the Log Rank test within the Kaplan–Meier analysis (Fig. 3A, B). Co-occurrence of high progenitor B-cell (>1.75%) and lymphocyte (>9.4%) percentages (*n* = 30) identified non-del(5q) patients that obtained HI-E in 63%, while non-del(5q) patients with lymphocytes and progenitor B-cell percentages below the optimized cut-offs (*n* = 26) obtained HI-E in only 8% (*p* < 0.0001; Fig. 3C).

### Mutational analysis

**Fig. 4:**
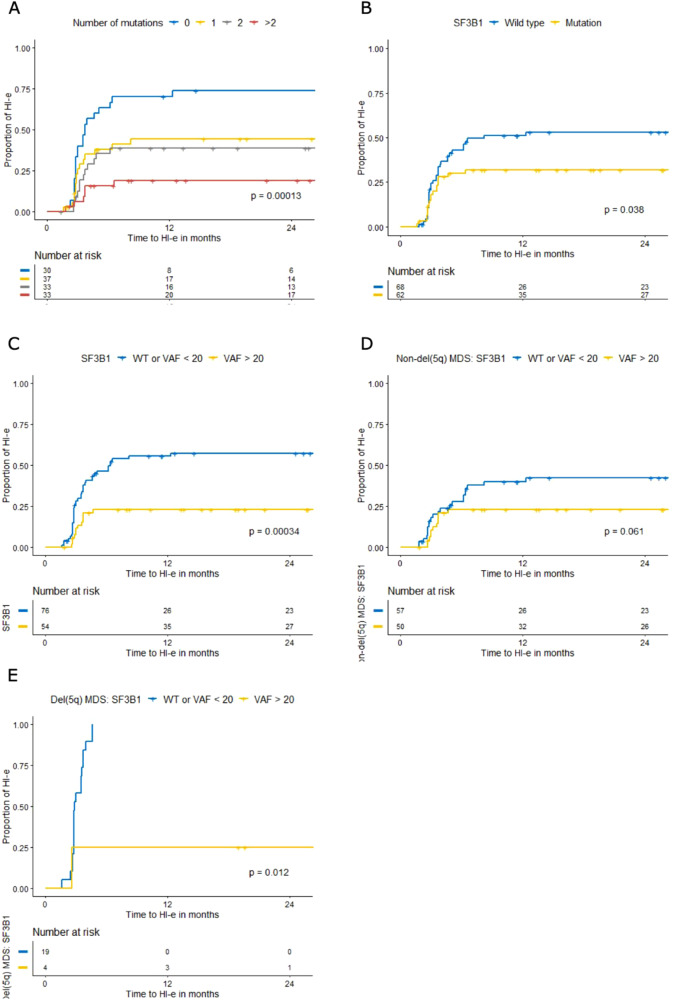
Determinants of response to lenalidomide by next-generation sequencing. Kaplan–Meier estimated erythroid hematological Improvement (HI-E) according to IWG2006 by **A** number of mutations; **B** SF3B1 mutation; **C** VAF percentages i.e., < or >20% of SF3B1 mutation in non-del(5q) and del(5q) MDS; **D** VAF percentage i.e., < or ≥20% of SF3B1 mutation in non-del(5q) MDS only and **E** VAF percentage i.e. < or ≥20% of SF3B1 mutation in MDS del(5q).

In 133/141 patients (94%) material was available for NGS. A total of 293 mutations in 15 of 19 myeloid genes investigated was found (Fig. S6). Most patients had no (22.6%), 1 (27.8%), or 2 mutations (24.8%) (Table S6a). The distribution of mutations per patient is shown in Fig. S6. *SF3B1* was most frequently affected (47.7%), followed by *TET2* (26.2%) and *ASXL1* (30.0%). *TP53* mutations, associated with lenalidomide resistance [10] were only present in one del(5q), and two non-del(5q) patients. The number of mutations within a patient was inversely correlated with HI-E (Fig. 4A, Fig. S7a, b, and Table S6b); no mutation: 70% vs. 1 mutation: 44% vs. 2 mutations 39% vs. >2 mutations 19%, *p* < 0.001 and had a negative impact on OS in non-del5(q) (Table S6c, *p* = 0.0074). Non-del(5q) showed higher numbers of mutations compared to del(5q) (no mutation 18% vs. 44%, 1 mutation 26% vs. 39%, 2 mutations 27% vs. 13%, >2 mutations 29% vs. 4%, *p* = 0.005, respectively).

In univariate analysis, several mutations significantly associated with HI-E, OS or PFS (Table S6b, c). *EZH2, SRSF2* and *SF3B1* mutations were inversely correlated with reaching HI-E. The presence of a *SF3B1* mutation was a negative predictor of response to lenalidomide (Fig. 4B; HI-E 31% vs. 53%, *p* = 0.038), especially at a VAF ≥ 20% (Fig. 4C, Supplementary Table S7b; HI-E 23% vs. 57%, *p* < 0.001). This effect of *SF3B1* mutation was present in both non-del(5q) (Fig. 4D, Table S6b, *p* = 0.061) as well as in del(5q) MDS (Fig. 4E, Table S6b, *p* = 0.012). All patients with a del(5q) and *SF3B1WT* or *SF3B1* mutation with a VAF < 20% reached HI-E, while only 1 out of 4 of the del(5q) patients with *SF3B1* mutation at a VAF > 20% reached HI-E upon lenalidomide treatment. This did not translate into a significant impact on OS or PFS (Table S6c). None of the patients with an EZH2 mutation (*n* = 7) showed HI-E, independently of VAF. For SRSF2 mutations (*n* = 11) there was only 1 patient who reached HI-E, this patient had a VAF of 21%. When combining all NGS parameters into a multivariate model, carrying del(5q) (HR 4.7 CI 2.62–8.59, *p* < 0.0001) and absence of a *SF3B1* mutation at a VAF > 20% independently predicted response to lenalidomide (HR 0.3, CI 0.19–0.72, *p* = 0.003). Within the non-del(5q) group, a low number of mutations was an independent predictive factor for response (HR 1.541, CI 1.13–2.11, *p* = 0.007), whereas in del(5q), the absence of a *SF3B1* mutation at a VAF > 20% was an independent predictor of response (HR 0.10, CI 0.01–0.81, *p* = 0.030).

### Flow cytometry and mutational analysis

To identify the most informative FC and mutational parameters for response to lenalidomide we combined the data in a multivariate model in a forward step manner. Carrying del(5q) was the most prominent predictor for response to lenalidomide (HR 3.67 CI 1.92–7.01, *p* = 0.0001). Higher percentages of lymphocytes (HR 1.05 CI 1.025–1.082, *p* = 0.0002) and a lower number of mutations (HR = 0.73, CI 0.546–0.988, *p* = 0.041) were independent prognostic factors for response for the entire group. In del(5q), high percentages of BM lymphocytes (HR 1.135, CI 1.058–1.218, *p* < 0.001) were independent predictors of response; within non-del(5q), higher percentages of lymphocytes and progenitor B-cells (HR 1.04, CI 1.005–1.073, *p* = 0.02 and HR 1.023, CI 1.001–1.046, *p* = 0.04, respectively).

## Discussion

In this randomized phase-II study in patients with lower-risk MDS including 84% of non-del(5q) MDS, HI-E was achieved in 39 and 41% of patients receiving lenalidomide without or with ESA/G-CSF, respectively. TI was achieved in 37% of patients, similar in both arms. MDS with del(5q) was included since at the date of start of the study lenalidomide was not yet registered for MDS del(5q). HI-E was significantly lower in non-del(5q) than in del(5q): 32% vs. 80%, respectively; the same accounted for TI: 30% vs. 73%, and TI at week 24: 16% and 67%, respectively. These findings are consistent with the phase-III MDS-005 study comparing lenalidomide vs. placebo in transfusion-dependent patients with low risk non-del(5q) MDS: HI-E in 36.5%, TI in 30% and TI at 24 weeks in 17.5% of patients [6]. Regarding MDS with del(5q), our data confirm the findings of the phase-II MDS-003 trial showing HI-E of 67%, the phase-III MDS-004 trial showing TI for 26 weeks or longer in 56.1% and the LE-MON5 with 67% of TI [24–26]. We observed no differences in PFS and OS between arms, nor between patients with or without del(5q). Patients who did respond to lenalidomide showed a significantly higher PFS and OS, similar between arms and between non-del(5q) and del(5q) MDS.

Previous studies showed that lenalidomide improves EPO signaling and restores the sensitivity of erythroid progenitors to ESA [11]. Toma showed in non-del(5q), that combination of lenalidomide/ESA significantly improved HI-E after 4 cycles (39.4%) as compared to lenalidomide alone (23.1%; *p* = 0.044). TI and response duration improved: 24.2% vs. 13.8% (*p* = 0.13) and 18.1 vs. 5.1 months, respectively, for lenalidomide/ESA vs. lenalidomide [7]. List demonstrated significantly higher major erythroid responses in non-del(5q) after 4 cycles of lenalidomide/ESA vs. lenalidomide: 28.3% vs. 11.5%, respectively [8]. In these studies lenalidomide/ESA were started simultaneously whereas in our study, a step-wise addition of ESA/G-CSF was given only in patients without response after 4 cycles of lenalidomide. No clear differences in baseline characteristics are found between the HOVON89 study and these studies. As expected, no differences in HI-E between arm A and B were observed after 4 cycles before ESA was added in arm B. However, we did not observe improvement of HI-E upon ESA exposure. This may indicate that upfront treatment with lenalidomide/ESA is more beneficial for reaching HI-E than a delayed ESA exposure. Unexpectedly, we observed a significant loss of patients in the first 4 cycles of lenalidomide in arm B vs. A. We could not explain these differences. Inexperience with lenalidomide in MDS might explain loss of patients in the first cycles. Differences between treating physicians were not obvious. Results were analyzed using the IWG2006 criteria, adhering to the statistical plan at study design. During this study, updated IWG response criteria were published (IWG2018) [27]. In this guideline, a response is defined if it is last for at least 16 weeks as compared to a 16 weeks screening period. Treatment steps in the experimental arm of our study were dictated by a 8-week response evaluation. Unfortunately, our dataset is incomplete and not suitable to evaluate response according to the latest IWG2018 criteria [27].

Our data do not support identified predictive markers for HI-E upon lenalidomide such as baseline EPO level, duration of MDS prior to study entry, previous treatment with ESA and IPSS risk [6, 28]. This is partly consistent with studies of Toma [7] and List [8], in which gender, age, WHO classification, IPSS and failure to respond to ESA did not predict response. Toma did show that baseline EPO level <100 U/L and pretreatment transfusion load were associated with better responses to lenalidomide/ESA [7].

FC evaluation of BM showed that patients responding to lenalidomide harbor higher lymphocyte and progenitor B-cell percentages, lower myeloid progenitor and neutrophil percentages. These data support findings from Kerdival who showed that progenitor B-cell and T cell-associated gene expression is enriched in non-del(5q) MDS that respond to lenalidomide [29]. The immune modulatory action of lenalidomide [13] may be enhanced with higher lymphocyte counts. High progenitor B-cell and low myeloid progenitor cell percentages, also associated with prolonged OS, reflecting a more normal BM cell composition in MDS [30]. These subsets are part of routine FC assessment of BM in patients suspected for MDS [31]. Further studies are required to validate the prediction model for non-del(5q) MDS in an independent dataset.

Our data confirm that number of myeloid driver mutations in MDS inversely correlates with response to lenalidomide [8, 14, 15]. Response to lenalidomide increased significantly in absence of driver mutations reaching 100% HI-E in del(5q) and over 55% in non-del(5q) MDS. Most mutations had no impact on achieving HI-E except for *SRSF2*, *EZH2* and *SF3B1* that correlated with a poor response to lenalidomide. *SF3B1* is frequently mutated in lower-risk MDS [32]. We identified *SF3B1* mutations in both del(5q) and non-del(5q) as negative predictor for response. *SF3B1* mutations at a VAF ≥ 20% were related to lack of response in non-del(5q) and in del(5q), while *SF3B1WT* or VAF < 20% showed a 42 and 100% response, respectively. Since *SF3B1* mutations are highly associated with the presence of ring sideroblasts [32], the absence of ring sideroblasts also predicted response to lenalidomide, though the latter contradicts other studies [7, 8]. Our observations on SF3B1 are in line with a report showing that *SF3B1* and *TET2* mutations were overrepresented in lenalidomide-refractory non-del(5q) MDS [14]. Another study identified *SF3B1* mutations as a poor prognostic marker in del(5q) MDS, although no association was made with lenalidomide responses [33]. The observation that *SF3B1* mutations with a lower VAF had no significant impact on response to lenalidomide may be explained by the fact that *SF3B1* is frequently affected in clonal hematopoiesis of indeterminate potential, a condition increasingly occurring in elderly people with mutations at diverse VAFs [34] The observation of non-responsiveness to lenalidomide in *SF3B1*-mutant del(5q) MDS is of clinical importance. It was shown that 59% of non-del(5q) patients with ring sideroblasts (of which 93% with *SF3B1* mutations) treated with luspatercept did show erythroid improvement [35]. It would be interesting to study patients with del(5q) and a *SF3B1* mutation [36], who do not achieve HI-E with lenalidomide, but may benefit from luspatercept as first-line treatment. Efficacy of lenalidomide was reported in MDS/MPN-RS-T with HI-E responses up to 53% [37]. As most of these patients harbor a SF3B1 mutation, correlation between SF3B1 mutations and a poor lenalidomide response cannot be generalized. Apart from a SF3B1 mutation, MDS/MPN-RS-T patients often carry other mutations which might modulate sensitivity to lenalidomide. In a recent report, 19 *SF3B1-*positive MDS/MPN-RS-T patients were treated with lenalidomide of whom 9 showed a response. Interestingly, in 5/9 responders, also a JAK2 mutation was found, whereas in only 2/10 of the non-responders a combination of SF3B1 and JAK2 was present. Larger series are necessary to establish a possible role of SF3B1 co-mutations on the responsiveness to lenalidomide.

The HOVON89 study confirms that a considerable number of ESA-refractory, transfusion-dependent low risk MDS patients may benefit from lenalidomide. We did not show additional effects of ESA/G-CSF on HI-E and TI when given sequentially if not responsive to lenalidomide. *SF3B1* mutations, del(5q) status, percentages of lymphocyte and progenitor B-cells and number of mutations proved predictive for reaching HI-E. Genotype and phenotype are closely related as reflected in the complementary value of the NGS and FC [38–40]. MDS harboring *SF3B1* mutations have lower lymphocyte and progenitor B-cell percentages as compared to MDS with *SF3B1WT* illustrating the relevance of factors predicting HI-E to lenalidomide. This clearly shows that treatment of low risk MDS can be guided by implementing predictive markers for response to lenalidomide.

### Supplementary information

supplementary text

supplementary figures and tables

## Data Availability

The datasets generated and/or analyzed during the current study are available from the corresponding author on reasonable request.

## References

[1] Malcovati L, Hellstrom-Lindberg E, Bowen D, Ades L, Cermak J, Del Canizo C (2013). Diagnosis and treatment of primary myelodysplastic syndromes in adults: recommendations from the European LeukemiaNet. Blood.

[2] Jadersten M, Malcovati L, Dybedal I, Della Porta MG, Invernizzi R, Montgomery SM (2008). Erythropoietin and granulocyte-colony stimulating factor treatment associated with improved survival in myelodysplastic syndrome. J Clin Oncol.

[3] Hellström-Lindberg E, van de Loosdrecht A (2013). Erythropoiesis stimulating agents and other growth factors in low-risk MDS. Best Pract Res Clin Haematol.

[4] Park S, Greenberg P, Yucel A, Farmer C, O’Neill F, De Oliveira Brandao C (2019). Clinical effectiveness and safety of erythropoietin-stimulating agents for the treatment of low- and intermediate-1-risk myelodysplastic syndrome: a systematic literature review. Br J Haematol.

[5] Fenaux P, Giagounidis A, Selleslag D, Beyne-Rauzy O, Mufti G, Mittelman M (2011). A randomized phase 3 study of lenalidomide versus placebo in RBC transfusion-dependent patients with Low-/Intermediate-1-risk myelodysplastic syndromes with del5q. Blood.

[6] Santini V, Almeida A, Giagounidis A, Gröpper S, Jonasova A, Vey N (2016). Randomized phase III study of lenalidomide versus placebo in RBC transfusion-dependent patients with lower-risk non-del(5q) myelodysplastic syndromes and ineligible for or refractory to erythropoiesis-stimulating agents. J Clin Oncol.

[7] Toma A, Kosmider O, Chevret S, Delaunay J, Stamatoullas A, Rose C (2016). Lenalidomide with or without erythropoietin in transfusion-dependent erythropoiesis-stimulating agent-refractory lower-risk MDS without 5q deletion. Leukemia.

[8] List AF, Sun Z, Verma A, Bennett JM, Komrokji RS, McGraw K (2021). Lenalidomide-epoetin alfa versus lenalidomide monotherapy in myelodysplastic syndromes refractory to recombinant erythropoietin. J Clin Oncol.

[9] Krönke J, Udeshi ND, Narla A, Grauman P, Hurst SN, McConkey M (2014). Lenalidomide causes selective degradation of IKZF1 and IKZF3 in multiple myeloma cells. Science.

[10] Krönke J, Fink EC, Hollenbach PW, MacBeth KJ, Hurst SN, Udeshi ND (2015). Lenalidomide induces ubiquitination and degradation of CK1α in del(5q) MDS. Nature.

[11] McGraw KL, Basiorka AA, Johnson JO, Clark J, Caceres G, Padron E (2014). Lenalidomide induces lipid raft assembly to enhance erythropoietin receptor signaling in myelodysplastic syndrome progenitors. PLoS ONE.

[12] Basiorka AA, McGraw KL, De Ceuninck L, Griner LN, Zhang L, Clark JA (2016). Lenalidomide stabilizes the erythropoietin receptor by inhibiting the E3 ubiquitin ligase RNF41. Cancer Res.

[13] Semeraro M, Vacchelli E, Eggermont A, Galon J, Zitvogel L, Kroemer G (2013). Trial watch: lenalidomide-based immunochemotherapy. Oncoimmunology.

[14] Adema V, Palomo L, Toma A, Kosmider O, Fuster-Tormo F, Benito R (2020). Distinct mutational pattern of myelodysplastic syndromes with and without 5q- treated with lenalidomide. Br J Haematol.

[15] Santini V, Fenaux P, Giagounidis A, Platzbecker U, List AF, Haferlach T (2021). Impact of somatic mutations on response to lenalidomide in lower-risk non-del(5q) myelodysplastic syndromes patients. Leukemia.

[16] Vardiman JW, Harris NL, Brunning RD (2002). The World Health Organization (WHO) classification of the myeloid neoplasms. Blood.

[17] Cheson BD, Greenberg PL, Bennett JM, Lowenberg B, Wijermans PW, Nimer SD (2006). Clinical application and proposal for modification of the International Working Group (IWG) response criteria in myelodysplasia. Blood.

[18] Westers TM, Ireland R, Kern W, Alhan C, Balleisen JS, Bettelheim P (2012). Standardization of flow cytometry in myelodysplastic syndromes: a report from an international consortium and the European LeukemiaNet Working Group. Leukemia.

[19] Sandmann S, de Graaf AO, van der Reijden BA, Jansen JH, Dugas M (2017). GLM-based optimization of NGS data analysis: a case study of Roche 454, Ion Torrent PGM and Illumina NextSeq sequencing data. PLoS ONE.

[20] Arber DA, Orazi A, Hasserjian R, Thiele J, Borowitz MJ, Le Beau MM (2016). The 2016 revision to the World Health Organization classification of myeloid neoplasms and acute leukemia. Blood.

[21] Wells DA, Benesch M, Loken MR, Vallejo C, Myerson D, Leisenring WM (2003). Myeloid and monocytic dyspoiesis as determined by flow cytometric scoring in myelodysplastic syndrome correlates with the IPSS and with outcome after hematopoietic stem cell transplantation. Blood.

[22] Della Porta MG, Picone C, Pascutto C, Malcovati L, Tamura H, Handa H (2012). Multicenter validation of a reproducible flow cytometric score for the diagnosis of low-grade myelodysplastic syndromes: results of a European LeukemiaNET study. Haematologica.

[23] Cremers EMP, Westers TM, Alhan C, Cali C, Visser-Wisselaar HA, Chitu DA (2017). Implementation of erythroid lineage analysis by flow cytometry in diagnostic models for myelodysplastic syndromes. Haematologica.

[24] List A, Dewald G, Bennett J, Giagounidis A, Raza A, Feldman E (2006). Lenalidomide in the myelodysplastic syndrome with chromosome 5q deletion. N Engl J Med.

[25] Giagounidis A, Mufti GJ, Mittelman M, Sanz G, Platzbecker U, Muus P (2014). Outcomes in RBC transfusion-dependent patients with Low-/Intermediate-1-risk myelodysplastic syndromes with isolated deletion 5q treated with lenalidomide: a subset analysis from the MDS-004 study. Eur J Haematol.

[26] Schuler E, Giagounidis A, Haase D, Shirneshan K, Büsche G, Platzbecker U (2016). Results of a multicenter prospective phase II trial investigating the safety and efficacy of lenalidomide in patients with myelodysplastic syndromes with isolated del(5q) (LE-MON 5). Leukemia.

[27] Platzbecker U, Fenaux P, Adès L, Giagounidis A, Santini V, van de Loosdrecht AA (2019). Proposals for revised IWG 2018 hematological response criteria in patients with MDS included in clinical trials. Blood.

[28] Santini V, Schemenau J, Levis A, Balleari E, Sapena R, Adès L (2013). Can the revised IPSS predict response to erythropoietic-stimulating agents in patients with classical IPSS low or intermediate-1 MDS?. Blood.

[29] Kerdivel G, Chesnais V, Becht E, Toma A, Cagnard N, Dumont F (2018). Lenalidomide-mediated erythroid improvement in non-del(5q) myelodysplastic syndromes is associated with bone marrow immuno-remodeling. Leukemia.

[30] Kahn JD, Chamuleau ME, Westers TM, Van de Ven PM, van Dreunen L, van Spronsen M (2015). Regulatory T cells and progenitor B cells are independent prognostic predictors in lower risk myelodysplastic syndromes. Haematologica.

[31] van de Loosdrecht AA, Kern W, Porwit A, Valent P, Kordasti S, Cremers EMP (2023). Clinical application of flow cytometry in patients with idiopathic cytopenias and suspected myelodysplastic syndrome: a report of the International Consortium and the European LeukemiaNet Working Group. Cytometry B Clin Cytom.

[32] Malcovati L, Stevenson K, Papaemmanuil E, Neuberg D, Bejar R, Boultwood J (2020). SF3B1-mutant MDS as a distinct disease subtype: a proposal from the International Working Group for the Prognosis of MDS. Blood.

[33] Meggendorfer M, Haferlach C, Kern W, Haferlach T (2017). Molecular analysis of myelodysplastic syndrome with isolated deletion of the long arm of chromosome 5 reveals a specific spectrum of molecular mutations with prognostic impact: a study on 123 patients and 27 genes. Haematologica.

[34] Jaiswal S, Ebert BL (2019). Clonal hematopoiesis in human aging and disease. Science.

[35] Fenaux P, Platzbecker U, Mufti GJ, Garcia-Manero G, Buckstein R, Santini V (2020). Luspatercept in patients with lower-risk myelodysplastic syndromes. N Engl J Med.

[36] Khoury JD, Solary E, Abla O, Akkari Y, Alaggio R, Apperley JF (2022). The 5th edition of the World Health Organization Classification of Haematolymphoid Tumours: myeloid and histiocytic/dendritic neoplasms. Leukemia.

[37] Komrokji R, Melody M, Al Ali N, Chan O, Klimek V, Ball BJ (2022). Treatment outcomes for patients with myelodysplastic syndrome/myeloproliferative neoplasms with ring sideroblasts and thrombocytosis. Leuk Lymphoma.

[38] Oelschlaegel U, Westers TM, Mohr B, Kramer M, Parmentier S, Sockel K (2015). Myelodysplastic syndromes with a deletion 5q display a characteristic immunophenotypic profile suitable for diagnostics and response monitoring. Haematologica.

[39] Duetz C, Westers TM, In ‘t Hout FEM, Cremers EMP, Alhan C, Venniker-Punt B (2021). Distinct bone marrow immunophenotypic features define the splicing factor 3B subunit 1 (SF3B1)-mutant myelodysplastic syndromes subtype. Br J Haematol.

[40] Weiß E, Walter W, Meggendorfer M, Baer C, Haferlach C, Haferlach T (2023). Identification of a specific immunophenotype associated with a consistent pattern of genetic mutations including SRFS2 and gene expression profile in MDS. Cytometry B Clin Cytom.

