# Supplementary material for: Determinants of lenalidomide response with or without erythropoiesis-stimulating agents in myelodysplastic syndromes: the HOVON89 trial
Source: Leukemia. 2024 Jan 31;38(4):840–50. doi: 10.1038/s41375-024-02161-6 (PMC10997501; doi:10.1038/s41375-024-02161-6)
Supplement: Supplementary file 1 — supplementary text [file 41375_2024_2161_MOESM1_ESM.pdf]

Supplementary TEXT HOVON89

Determinants of lenalidomide response with or without erythropoiesis-stimulating agents in myelodysplastic syndromes: the HOVON89 trial

A.A. van de Loosdrecht[1], E.M.P. Cremers[1,2,3]*, C. Alhan[1]*, C. Duetz[1]**, F.E.M. in 't Hout[2,3]**, H.A. Visser-Wisselaar[4], D.A. Chitu[4,8], A. Verbrugge[4], S.M. Cunha[4], G.J. Ossenkoppele[1], J.J.W.M. Janssen[1], S.K. Klein[5,6], E. Vellenga[6], G.A. Huls[6], P. Muus[3,7], S.M.C. Langemeijer[2,3], G.E. de Greef[8], P.A.W. te Boekhorst[8], M.H.G. Raaijmakers[8], M. van Marwijk Kooy[9], M.C. Legdeur[10], J.J. Wegman[11,12], W. Deenik[13], O. de Weerdt[14], T.M. van Maanen-Lamme[15], P. Jobse[16], R.J.W. van Kampen[17], A. Beeker[18], P.W. Wijermans[19], B.J. Biemond[12], B.C. Tanis[20], J.W.J. van Esser[21], C.G. Schaar[22], H.S. Noordzij-Nooteboom[23], E.M.G. Jacobs[24], A.O. de Graaf[2], M. Jongen-Lavrencic[8], M.J.P.L. Stevens-Kroef[25], T.M. Westers[1]***, J. H. Jansen[2]***, on behalf of the MDS HOVON89 study group.

Supplementary information on 'Patients and Methods'

Inclusion and exclusion criteria

Patients with MDS classified as RA, RARS, RCMD, RCMD-RS, RAEB-1, MDS-U and del(5q) according to WHO2001 [ref 1] with an IPSS ≤1.0 or patients with MDS/MPD (CMML-1 according to WHO, with a WBC≤12x10^9/L) were included in the study (table 1). Before study entry patients had to have Hb ≤6.2 mmol/L (10.0 g/dL) or Hb ≤7.2 mmol/L (11.6 g/dl) and absolute neutrophil count (ANC) ≤1.0x10^9/L or red blood cell transfusion-dependent at ≥2 units RBC during at least 8 weeks; units must be given for a Hb ≤5.6 mmol/L; 9 g/dl)). Patients were included with a WHO performance status 0-2 and with serum erythropoietin levels >200 U/L or ≤200 U/L and if failure of response or loss of hematological improvement or disease progression to maximal RAEB-1 after prior standard therapy with ESA/G-CSF. ESA/G-CSF had to be stopped at least 1 month before randomization. Central cytological and cytogenetic review were provided by Hematocytology Review Committee (HRC) of HOVON. Patients with severe cardiac, pulmonary, neurologic, metabolic or psychiatric diseases or active other malignancies were excluded for study enrollment as was anemia due to other causes than MDS including iron, vitamin B12 and folate deficiencies, auto-immune hemolysis and/or paroxysmal nocturnal hemoglobinuria (PNH) and hypoplastic MDS. Patients with active uncontrolled infection, an absolute neutrophil count (ANC) <0.5x10^9/L, platelet transfusion-dependent or with platelet counts <25x10^9/L or patients with active bleeding were excluded. Patients treated with biological response modifiers (i.e. growth factors, immunosuppressive agents and/or chemotherapy) within 1 month prior to randomization and prior treatment with lenalidomide were excluded.

Study Objectives and Endpoints

The primary objective of the study was to evaluate the efficacy of lenalidomide with or without treatment with ESA/G-CSF in terms of Erythroid response (HI-E) and HI defined by the IWG response criteria [2]. The secondary objectives were safety and tolerability, time-to-HI(-E), duration-of-HI(-E), number of given treatment cycles per patient, number of patients receiving ESA/G-CSF for arm B, response rate, progression free survival (PFS), overall survival (OS) and RBC transfusion requirements. Time-to-HI-E was defined as time between start protocol treatment and confirmation of HI-E. Duration-of-HI-E and duration-of-HI were defined as time from confirmation of HI-E or HI to relapse (loss of HI-E or HI). When no relapse was recorded, and patients were still on protocol, duration-of-HI-E and duration-of-HI was computed until date of last contact. PFS was defined as the time from registration to relapse or disease progression (IWG2006 [ref 2] or death, whichever came first. OS was defined as time from registration until death from any cause. The primary endpoint of the study was HI-E and HI according to IWG2006 [ref 2]. HI concerns three separate responses of cytopenias: erythroid, platelet and neutrophil response. A patient was classified as responder (any type of response) if the response was reached during the induction cycles. All other patients were classified as non-responders. Responses must last at least 8 weeks. HI-E for patients with pretreatment Hb < 6.8 mmol/L; 11 g/dL) was defined as either an Hb increase by ≥ 0.9 mmol/L (1.5 g/dL) or relevant reduction of units of RBC transfusions by an absolute number of at least 4 RBC transfusions per 8 weeks compared with the pretreatment transfusion number in the previous 8 weeks. Platelet response (pretreatment <100 x 10^9/L) was defined as absolute increase of ≥30 x10^9/L for patients starting with >20 x10^9/L platelets or increase from <20 x10^9/L to >20 x10^9/L and by at least 100%. Neutrophil response (pretreatment <1.0 x10^9/L) was defined as at least 100% increase and an absolute increase >0.5 x10^9/L. The primary endpoint was HI-E and HI according to IWG2006 criteria [2].

Treatment schedule arm A and arm B

The dosing regimen for lenalidomide in arm A was 10 mg once daily, orally on days 1-21 every 28 days. When tolerated, this dosing regimen was continued for at least 6 cycles. If no HI according to the modified IWG response criteria for MDS was obtained after 6 cycles, the patient went off-protocol [2]. If HI was reached after 6 cycles, the patient received another two cycles of lenalidomide until relapse. If no relapse/loss of response after 12 cycles, the patient continued with maintenance cycles of lenalidomide on the same dose as the last cycle until disease progression or transfusion requirement returned to baseline. The dosing regimen for lenalidomide in arm B was 10 mg once daily, orally on days 1-21 every 28 days for the first 4 cycles. If after cycle 4 HI-E according to the modified IWG response criteria for MDS was reached, the patient continued for cycles with lenalidomide monotherapy. If no HI was obtained

after 4 cycles of lenalidomide, ESA (NeoRecormon™) was started by s.c. administration at a dose of 30,000 IU weekly for 2 cycles (5th and 6th cycle). The dose of ESA (NeoRecormon™) was increased to 60,000 IU weekly if no HI was obtained after 2 cycles with ESA (7th and 8th cycle). If no HI was reached after 2 cycles of 60,000 IU ESA weekly, G-CSF (Neupogen™) was administrated at cycle 9-12. G-CSF was administrated 3 times weekly (3x300 µg/week s.c. for patients ≤ 75kg; 3x480 µg/week s.c. for patients > 75 kg). Treatment continued for at least 12 cycles for arm B. If no HI was obtained after 12 cycles, the patient went off-protocol. Otherwise, patients continued with maintenance cycles of lenalidomide with or without ESA/G-CSF at the dose level reached after 12 cycles. Dose modifications for lenalidomide, ESA/G-CSF were followed according to previously described guidelines (see supplement 1, protocol).

Statistical Methods

All analyses were performed according to the intention-to-treat principle. Patients initially randomized but who appeared to be ineligible based on information that should have been available at the date of inclusion were excluded from all analyses. The following decision rules regarding efficacy were specified in the protocol: i. stop because of inefficacy in case arm B would in truth be less or as effective as arm A (i.e. true difference in response rate arm B minus arm A ≤0) or in case no difference in response rate in favor of arm B was to be expected (i.e. upper limit of 80% confidence interval (CI) of the difference in response rate is <20%); ii. continue in a phase-III; iii. otherwise continue.

Biomarker studies

Flow cytometry

Bone marrow aspirates were processed within 24 hours. Mature erythrocytes were lysed using ammonium chloride-based erythrocyte lysing solution. Nucleated cells were pre-incubated with human serum immunoglobulins before staining. The antibody panels are provided in the supplementary file. A 4-color method was applied between 2009 and 2012 (FACS Calibur; BD Biosciences, San Jose, CA), and due to technical developments, an 8-color method (FACS Canto II; BD Biosciences) between 2012 and 2015. A minimum of 100,000 leukocyte events were acquired per antibody combination, including a minimum of 250 CD45diminished/CD34+ events. Data from the 4-color method were analyzed with CellQuestPro software (BD Biosciences); data from the 8-color method were analyzed with Infinicyt software (Cytognos, Salamanca, Spain). Results were discussed within an expert MDS-flow cytometry team, with ≥3 researchers to establish the Ogata score, integrated flow cytometry score (iFS) and the FCSS [3, 4, 5)]. Reference values for marker expression were established within normal, age-matched controls; values beyond two-times the standard deviation from normal are considered aberrant. Beyond the flow cytometry scores, we analyzed bone marrow cell subset

composition, including the percentages of the following cell populations: lymphocytes, neutrophils, monocytes, basophils, mast cells and plasmacytoid dendritic cells expressed as a percentage of total bone marrow white blood cells, progenitor-B cells as a percentage of CD34 positive cells, nucleated erythroid cells and myeloid progenitors as a percentage of all nucleated cells and CD117-positive erythroid cells as a percentage of nucleated erythroid cells.

NGS data processing and variant calling

Analysis was performed as described by Sandmann et al. [6]. Processing is described in short below.

Ion Torrent Sequencing

Amplicons covering relevant regions of the genes of interest were sequenced using Ion Torrent semiconductor technology, as described. PCRs were performed using conventional sequencing primers in a fully automated robotic work flow. PCR amplicons were subsequently pooled and sheared to 200-300 bp. Library preparation was performed in an automated fashion on a MicroLab Starlet Replicator Robot (Hamilton) by using the Ion Plus fragment library kit in combination with the Ion XpressTM barcode adapters 1-96 kit (both Life Technologies). Emulsion PCRs were performed on an Ion OneTouch system (Ion OT2 instrument, Life Technologies) using the Ion PGM Template OT2 200 kit. Enrichment of template-positive Ion sphere particles (ISPs) was performed on a OneTouch ES system (Life Technologies). The percentage of template-positive ISPs was measured with use of the Ion Sphere Quality Control kit (Life Technologies) and a Qubit 2.0 Fluorometer (Invitrogen). Subsequently, ISPs coated with template were loaded on Ion 318TM sequencing chips (Life Technologies). The chips were sequenced on the PGM, using the Ion PGM sequencing 200 kit version 2 (Life Technologies).

Illumina NextSeq Sequencing

The genomic library was prepared using 50 ng of DNA template and TruSight DNA Amplicon Sequencing Panel Library Prep Kit (Illumina, San Diego, CA) according to the manufacturer's protocol. Briefly, library preparation involved hybridization of the probe mixture to genomic DNA and areas of interest were captured by extension and ligation. In addition to areas complimentary to genomic DNA, each of the probes has a common sequence, which is used to PCR-amplify the captured sequences in a subsequent step. This PCR step also adds sequencing adapters (for binding of sequencing primers) and short stretches of identifier sequences or barcodes on the 3' and 5' ends of the amplicons to be used as unique sample identifiers, thus facilitating multiplexed sequencing of the samples. The library generated using the TruSight Myeloid Sequencing Panel was purified using AMPure magnetic beads (Agentcourt, Brea, CA) according to the manufacturer's protocol. From each library, equal quantities of the DNA were eluted using Library normalization beads (TruSight kit) following the manufacturer's instructions and equal volumes were mixed. This ensures similar representation of the library from each sample during multiplexed sequencing. Paired end sequencing of samples was performed using NextSeq 500 mid output Reagent Kit, V2 (2 x 150 cycles) using the NextSeq 500 sequencer (Illumina). On average, libraries from 32

samples per sequencing run were multiplexed. BCL data produced by the NextSeq 500 instruments were converted into demultiplexed fastq files using bcl2fastq 2.14 (Illumina).
Bcl to fastq conversion and demultiplexing of barcoded reads was performed automatically and data was uploaded to a server running commercial analysis software (Sequence Pilot, JSI medical systems, Ettenheim, Germany). The following settings were used for variant calling using Sequence Pilot:

(i) Required Coverage/Min abs. cov., 20 combined

(ii) Mutations/Min abs. cov., 10 combined

(iii) Min % cov., 5% per dir

After variant calling using the commercial software, all variants were manually inspected and curated
using in-house databases for recurrent artifacts, polymorphisms and mutations. Furthermore, Alamut Visual (Interactive Biosoftware, Rouen, France) and publicly available databases such as dbSNP, COSMIC, ClinVar, gnomAD, ESP were used to identify rare polymorphisms, which were excluded.

Supplementary Results

Patients (see table 1)

Sixteen patients were ineligible. Seven patients were at higher risk IPSS 1.5 and two patients were not treated with standard ESA/G-CSF before study entry. One patient appeared to be not lenalidomide-naïve. In three patients the diagnosis of MDS could not be confirmed, one patient suffered from a second active malignancy, one patient had ANC counts <0.8 at study entry and one patient was registered twice. These patients were not included in the final analysis.

Baseline characteristics were comparable among treatment arms, including age, gender, blood cell counts, WHO performance, WHO diagnosis (including del(5q)), IPSS, previous treatments, and endogenous EPO levels. Patient characteristics are summarized in table 1.

No HI-E was observed in 51% of those patients not previously exposed to ESA and in 64% of those exposed to ESA/G-CSF prior to study entry. This was not significantly different between groups (p=0.094) nor differences in HI-E between arms were found in either previously or not previously exposed to ESA/G-CSF (p=0.600 and p=0.480, respectively).

Impact of WHO2016-MDS del(5q) definition.

Since we designed the HOVON89 study before the implementation of the WHO2016 classification, we reclassified the patients with chromosome 5 abnormalities according to WHO2016 definitions [7]. Hence three additional patients were classified as MDS del(5q); all were included in arm A (n=13->16), arm B n=17, unchanged). Seventy-nine percent of the WHO-2016-del (5q) patients achieved HI-E similar as compared to the MDS del (5q) defined by the WHO2001. In the non-del(5q) group (n=151), HI-E was 32%. Again, transfusion independency (TI) differed significantly at week 24; 64% for the MDS del(5q) vs. 15% for the non-del(5q), but we observed no significant differences in PFS and OS similar to the definitions of WHO2001 [1].

References

1. Vardiman JW, Harris NL, Brunning RD. The World Health Organization (WHO) classification of the myeloid neoplasms. Blood. 2002;100:2292-302.
2. Cheson BD, Greenberg PL, Bennett JM, Lowenberg B, Wijermans PW, Nimer SD, et al. Clinical application and proposal for modification of the International Working Group (IWG) response criteria in myelodysplasia. Blood. 2006;108:419-25.
3. Della Porta MG, Picone C, Pascutto C, Malcovati L, Tamura H, Handa H, et al. Multicenter validation of a reproducible flow cytometric score for the diagnosis of low-grade myelodysplastic syndromes: results of a European LeukemiaNET study. Haematologica. 2012;97:1209-17.
4. Alhan C, Westers TM, Cremers EMP, Cali C, Ossenkoppele GJ, van de Loosdrecht AA. Application of flow cytometry for myelodysplastic syndromes: Pitfalls and technical considerations. Cytometry B Clin Cytom. 2016;90:358-67.
5. Cremers EMP, Westers TM, Alhan C, Cali C, Visser-Wisselaar HA, Chitu DA, et al. Implementation of erythroid lineage analysis by flow cytometry in diagnostic models for myelodysplastic syndromes. Haematologica. 2017;102:320-6.
6. Sandmann S, de Graaf AO, van der Reijden BA, Jansen JH, Dugas M. GLM-based optimization of NGS data analysis: A case study of Roche 454, Ion Torrent PGM and Illumina NextSeq sequencing data. PLoS One. 2017;12:e0171983.
7. Arber DA, Orazi A, Hasserjian R, Thiele J, Borowitz MJ, Le Beau MM, et al. The 2016 revision to the World Health Organization classification of myeloid neoplasms and acute leukemia. Blood. 2016;127:2391-405.
