# Supplementary material for: Determinants of lenalidomide response with or without erythropoiesis-stimulating agents in myelodysplastic syndromes: the HOVON89 trial
Source: Leukemia. 2024 Jan 31;38(4):840–50. doi: 10.1038/s41375-024-02161-6 (PMC10997501; doi:10.1038/s41375-024-02161-6)

Supplementary figures and tables HOVON89

Contents:

Table S1a-b: Flow cytometry panels

Table S2: NGS panel

Table S3a: Proportion of patients with adverse events of CTCAE grade 2-4 per treatment arm for cycles 1-12.

Table S3b: Proportion of patients with adverse events of CTCAE grade 2-4 per treatment arm for cycles 1-4.

Table S4: Responses according to IWG2006 in non-del(5q) MDS: non-RS vs. RS (WHO2001)

Table S5: HI-E and duration of HI-E by FC-defined parameters as predictors of response.

Table S6a: Number of genes mutated in patients with non-del(5q) and del(5q) MDS.

Table S6b: NGS-defined parameters as predictors of HI-E and duration.

Table S6c: NGS-defined parameters as predictors of OS and PFS.

Figure S1: Randomization scheme of HOVON89 arm A vs. arm B.

Figures S2: Leukemic evolution according to arm A and arm B.

Figures S3a: OS stratified for pretreament with ESA/G-CSF (fig. S3a).

Figures S3b-c: OS and PFS according to IPSS risk (fig. S3b-c).

Figures S4a-b: PFS and OS for non-del(5q) and del(5q) MDS in HOVON89

Figures S5a-c: Landmark analysis for OS according to HI-E.

Figure S5a: OS of all MDS patients.

Figure S5b: OS of MDS del(5q).

Fig. S5c: OS of MDS non-del(5q).

Figure S6: Overview of mutations and co-mutations per patient.

Figures S7a-b: HI-E in non-del(5q) and del(5q) according to number of mutations.

Table S1: Flow cytometry panels (HOVON89)

*a. **4-color flow cytometry panel***

	FITC	PE	PerCP	APC
**1**	-	-	CD45 (2D1) BD	-
**2**	CD16 (DJ130c) DAKO	CD13 (L138) BD	CD45 (2D1) BD	CD11b (D12) BD
**3**	CD34 (8G12) BD	CD11b (D12) BD	CD45 (2D1) BD	-
**4**	CD36 (CLB-IVC7) Sanquin	CD33 (P67.6) BD	CD45 (2D1) BD	CD11b (D12) BD
**5**	CD36 (CLB-IVC7) Sanquin	CD64 (10.1) DAKO	CD45 (2D1) BD	HLA-DR (L243) BD
**6**	CD15 (MMA) BD	CD10 (SS2/36) DAKO	CD45 (2D1) BD	CD14 (MoP9) BD
**7**	CD34 (8G12) BD	CD117 (104D2) BD	CD45 (2D1) BD	CD13 (WM15) CD33 (P67.6) both BD
**8**	-	-	CD45 (2D1) BD	CD34 (8G12) BD
**9**	CD5 (CK23) DAKO	CD19 (SJ25C1) BD	CD45 (2D1) BD	CD34 (8G12) BD
**10**	CD2 (MT910) DAKO	CD56 (My31) BD	CD45 (2D1) BD	CD34 (8G12) BD
**11**	CD13 (WM-47) DAKO	CD7 (M-T701) BD	CD45 (2D1) BD	CD34 (8G12) BD
**12**	CD13 (WM-47) DAKO	CD25 (ACT-1) DAKO	CD45 (2D1) BD	CD34 (8G12) BD
**13**	CD71 (Ber-T9) BD	CD235a (JC159) DAKO	CD45 (2D1) BD	CD117 (103D2) DAKO

*b. **8-color flow cytometry panel***

	FITC	PE	PerCP-Cy5.5	PC7	APC	APC-H7	HV450	KO
**1**	-	-	CD34 (8G12) BD	CD117 (104D21) BC	-	-	HLA-DR (L243) BD	CD45 (J.33) BC
**2**	CD16 (DJ130c) DAKO	CD13 (L138) BD	CD34 (8G12) BD	CD117 (104D21) BC	CD11b (D12) BD	CD10 (HI1A) BD	HLA-DR (L243) BD	CD45 (J.33) BC
**3**	CD2 (MT910) DAKO	CD64 (10.1) DAKO	CD34 (8G12) BD	CD117 (104D21) BC	IREM2 (UP0H2) IS	CD14 (MoP9) BD	HLA-DR (L243) BD	CD45 (J.33) BC
**4**	CD36 (CLB-IVC7) Sanquin	CD105 (43A3) BL	CD34 (8G12) BD	CD117 (104D21) BC	CD33 (P67.6) BD	CD71 (M-A712) BD	HLA-DR (L243) BD	CD45 (J.33) BC
**5**	CD5 (CD23) DAKO	CD56 (My31) BD	CD34 (8G12) BD	CD117 (104D21) BC	CD7 (M-T701) BD	CD19 (SJ25C1) BD	HLA-DR (L243) BD	CD45 (J.33) BC
**6**	CD15 (MMA) BD	CD25 (ACT-1) DAKO	CD34 (8G12) BD	CD117 (104D21) BC	CD123 (9F5) BD	CD38 (HB7) BD	HLA-DR (L243) BD	CD45 (J.33) BC
**7**	CD7 (M-T701) BD	CD235a (JC159) DAKO	CD34 (8G12) BD	CD117 (104D21) BC	CD13 (WM15) BD	CD71 (M-A712) BD	HLA-DR (L243) BD	CD45 (J.33) BC

Note: Per antibody CD number, clone and manufacturer are indicated. BC: Beckmann Coulter, Miami, FL; BD: Becton Dickinson, San Jose, CA; BL: Biolegend, San Diego, CA; DAKO, DakoCytomation, Glostrup, Denmark; IS: Immunostep, Salamanca, Spain; Sanquin, Amsterdam, The Netherlands.

Table S2: Sequencing panel used for HOVON89

Gene	Exons	TranscriptID
ASXL1	E13	ENST00000375687
CBL	E8, E9	ENST00000264033
DNMT3A	E2-E23	ENST00000264709
ETV6	E1-E8	ENST00000396373
EZH2	E2-E20	ENST00000320356
FLT3	E20	ENST00000241453
IDH1	E4	ENST00000345146
IDH2	E4	ENST00000330062
JAK2	E12, E14	ENST00000381652
KRAS	E2, E3	ENST00000256078
NPM1	E11	ENST00000296930
NRAS	E2, E3	ENST00000369535
RUNX1	E3-E8	ENST00000437180
SF3B1	E13, E14, E16	ENST00000392485
SRSF2	E1	ENST00000392485
TET2	E3-E11	ENST00000380013
TP53	E2-E11	ENST00000269305
U2AF1	E2, E6	ENST00000291552
ZRSR2	E1-E11	ENST00000307771

Table S3a: Proportion of patients with adverse events of CTCAE grade 2-4 specified per treatment arm for cycles 1-12

	Arm A (n=92)			Arm B (n=91)*		
CTCAE	grade 2	grade 3	grade 4	grade 2	grade 3	grade 4
Any event	23 (25)	40 (43)	20 (22)	21 (23)	48 (53)	17 (19)
Blood/bonemarrow	14 (15)	18 (20)	11 (12)	14 (15)	17 (19)	8 (9)
Constitutional symptoms	21 (23)	8 (9)	-	23 (25)	9 (10)	1 (1)
Infections	11 (12)	11 (12)	1 (1)	18 (20)	14 (15)	5 (5)
Gastrointestinal	25 (27)	6 (7)	-	14 (15)	8 (9)	-
Dermatology/skin	22 (24)	4 (4)	4 (4)	15 (16)	6 (7)	-
Pulmonary	8 (9)	6 (7)	-	13 (14)	3 (3)	1 (1)
Neurology	7 (8)	5 (5)	-	10 (11)	4 (4)	1 (1)
Metabolic	3 (3)	6 (7)	-	12 (13)	3 (3)	1 (1)
Musculoskeletal	9 (10)	2 (2)	-	7 (8)	4 (4)	-
Pain	12 (13)	2 (2)	-	5 (5)	2 (2)	-
Cardiac general	2 (2)	4 (4)	1 (1)	2 (2)	5 (5)	2 (2)
Cardiac arrythmia	3 (3)	-	1 (1)	3 (3)	5 (5)	-
Renal/genitourinary	5 (5)	1 (1)	-	3 (3)	3 (3)	-
Lymphatics	-	-	-	5 (5)	2 (2)	-
Vascular	1 (1)	2 (2)	2 (2)	1 (1)	1 (1)	-
Hemorrhage/bleeding	2 (2)	-	-	2 (2)	-	1 (1)
Hepatobiliary/pancreas	1 (1)	1 (1)	-	1 (1)	1 (1)	-
Allergy/immunology	-	1 (1)	-	2 (2)	1 (1)	-
Coagulation	-	-	-	1 (1)	2 (2)	-
Secondary malignancy	-	1 (1)	2 (2)	-	-	-
Surgery	-	1 (1)	-	1 (1)	1 (1)	-
Auditory/ear	-	-	-	2 (2)	-	-
Endocrine	1 (1)	-	-	1 (1)	-	-
Ocular/visual	1 (1)	-	-	1 (1)	-	-

Note: Numbers and percentages (between brackets) are displayed. Per adverse event, the maximum observed grade per cycle within a patient is reported. *1 patient never started with lenalidomide. Abbreviations: CTCAE: common terminology for adverse events

Table S3b: Proportion of adverse events of CTCAE grade 2-3-4 specified per treatment arm for cycles 1-4

	Arm A			Arm B		
n	92			91*		
CTCAE	grade 2	grade 3	grade 4	grade 2	grade 3	grade 4
Any event, number (%)	28 (30)	37 (40)	10 (11)	29 (32)	36 (40)	15 (16)
Blood/bonemarrow	11 (12)	18 (20)	5 (5)	9 (10)	10 (11)	8 (9)
Constitutional symptoms	17 (18)	8 (9)	-	18 (20)	6 (7)	1 (1)
infections	9 (10)	9 (10)	-	13 (14)	8 (9)	5 (5)
Gastrointestinal	17 (18)	5 (5)	-	11 (12)	5 (5)	-
Dermatology/skin	19 (21)	4 (4)	3 (3)	12 (13)	5 (5)	-
Pulmonary	5 (5)	2 (2)	-	11 (12)	3 (3)	1 (1)
neurology	4 (4)	4 (4)	-	5 (5)	3 (3)	-
metabolic	5 (5)	4 (4)	-	10 (11)	2 (2)	1 (1)
musculoskeletal	6 (7)	-	-	3 (3)	4 (4)	-
pain	9 (10)	-	-	4 (4)	2 (2)	-
Cardiac general	2 (2)	3 (3)	-	2 (2)	5 (5)	1 (1)
Cardiac arrythmia	3 (3)	-	-	2 (2)	5 (5)	-
Renal/genitourinary	2 (2)	1 (1)	-	3 (3)	3 (3)	-
lymphatics	-	-	-	2 (2)	1 (1)	-
vascular	-	2 (2)	2 (2)	1 (1)	1 (1)	-
Hemorrhage/bleeding	1 (1)	-	-	1 (1)	2 (2)	-
Hepatobiliary/pancreas	1 (1)	-	-	-	1 (1)	-
Allergy/immunology	-	1 (1)	-	1 (1)	1 (1)	-
coagulation	-	-	-	1 (1)	2 (2)	-
Secondary malignancy	-	1 (1)	-	-	-	-
Surgery	-	1 (1)	-	1 (1)	1 (1)	-
Auditory/ear	-	-	-	1 (1)	-	-
Ocular/visual	-	-	-	1 (1)	-	-

Note: Numbers and percentages (between brackets) are displayed. Per adverse event, the maximum observed grade per cycle within a patient is reported.*1 patient never started with treatment. Abbreviations: CTCAE: common terminology for adverse events

Table S4: Responses according to IWG2006 in non-del(5q) MDS: non-RS vs. RS (WHO2001)

	Non-RS	RS	Total cohort
**n**	79	75	154
**HI-E, n (%)**	32 (41%)	18 (24%)	50 (32%) *(p=0.029)*
**HI (HI-E/P/N), n (%)**	25 (32%)	18 (24%)	43 (28%)
**Time-to-HI-E, median months (range)**<br>**Time-to-HI-E, median weeks (range)**	2.9 (1.8-12.3)<br>11.7 (7.4-49.3)	3.6 (1.8-4.6)<br>14.5 (7.2-25.8)	3.2 (1.8-12.3)<br>12.9 (7.2-49.3)
**Time-to-HI (HI-E/P/N), median months (range)**	3.7 (1.8-11.0)	3.6 (1.8-6.4)	3.6 (1.8-11.0)
**Duration of HI-E, median months (range)**	8.9 (0.7-119)	7.4 (1.5-135)	8.3 (0.7-135)
**Duration of HI, median months (range)**	11.2 (0.7-119)	7.4 (1.5-135)	9.2 (0.7-135)
**HI-E at 24 weeks, n (%)**<br>**HI at 24 weeks n (%)**	19 (24%)<br>17 (22%)	10 (13%)<br>10 (13%)	29 (19%)<br>27 (18%)
**TI, n (%)**<br>**Time-to-TI, median months (range)**<br>**TI at 24 weeks, n (%)**	29 (37%)<br>2.9 (1.8-12.3)<br>14 (18%)	16 (21%)<br>3.4 (1.8-6.4)<br>10 (13%)	45 (29%) *(p=0.037)*<br>3.2 (1.8-12.3)<br>24 (16%)
**CR, CRi, CRd including TI, n (%)**<br>**SD (+ no response, n (%))**<br>**PD, n (%)**	21 (26%)<br>28 (35%) (+16, 20%)<br>14 (18%)	16 (22%)<br>35 (47%) (+20, 27%)<br>4 (5%)	37 (25%)<br>63 (41%) (+36, 23%)<br>18 (12%)

Abbreviations: CR: complete remission; CRi: complete remission with incomplete peripheral blood recovery; CRd: complete remission with persistence of dysplasia; HI: hematologic improvement; HI-E/P/N: hematologic improvement-erythroid/platelet/neutrophil; TI: transfusion-independency; MDS: myelodysplastic syndroms; RS: ring sideroblasts; n: number; PD: progressive disease; SD: stable disease

Table S5: HI-E and duration of HI-E by flow cytometry-defined parameters as predictors of response; OS and PFS by flow cytometry-defined parameters

	**HI_E**			**Duration HI_E**		
	**All MDS**	**Del(5q) MDS**	**non-del(5q) MDS**	**All MDS**	**Del(5q) MDS**	**non-del(5q) MDS**
**iFS**	n.s.	n.s.	n.s.	n.s.	n.s.	n.s.
**Ogata**	n.s.	n.s.	n.s.	n.s.	n.s.	n.s.
**FCSS**	n.s.	n.s.	n.s.	n.s.	n.s.	n.s.
**Progenitor B-cells**	HR 1.024 (CI 1.005 - 1.043), *p* = 0.013	n.s.	HR 1.03 (CI 1.009 - 1.052), *p* = 0.005	rho 0.31 (CI 0.04-0.54), *p* = 0.02	n.s.	rho 0.38 (CI 0.04-064), *p* = 0.03
**Myeloid progenitors**	n.s.	n.s.	HR 0.6928 (0.483-0.995), *p* = 0.047	n.s.	n.s.	n.s.
**Lymphocytes**	HR 1.064 (CI 1.04-1.09), *p* < 0.001	HR 1.135 (CI 1.058-1.218), *p* < 0.001	HR 1.046 (CI 1.014 - 1.078), *p* = 0.004	n.s.	n.s.	n.s.
**Neutrophils**	HR 0.96 (CI 0.9432-0.9767), *p* < 0.001	HR 0.94 (CI 0.908 - 0.974), *p* < 0.001	HR 0.97 (CI 0.949 - 0.992), *p* =0.008	n.s.	n.s.	n.s.
**Erytroid progenitors**	n.s.	n.s.	n.s.	n.s.	n.s.	n.s.
**pDCs**	n.s.	n.s.	n.s.	n.s.	n.s.	n.s.
**Basophils**	HR 1.43 (CI 1.097 - 1.862), *p* = 0.008	n.s.	n.s.	n.s.	n.s.	n.s.
**Eosinophils**	n.s.	n.s.	n.s.	n.s.	n.s.	n.s.
	**OS**			**PFS**		
	**All MDS**	**Del(5q) MDS**	**non-del(5q) MDS**	**All MDS**	**Del(5q) MDS**	**non-del(5q) MDS**
**iFS**	53% vs 23%, p = 0.001	n.s.	55% vs 19%, p < 0.001	p = 0.036	n.s.	p = 0.01
**Ogata**	52% vs 25%, p = 0.0032	n.s.	57% vs 19% p < 0.001	p = 0.0018	n.s.	p = 0.00029
**FCSS**	p < 0.0001	n.s.	p < 0.0001	p < 0.001	n.s.	p < 0.001
**Progenitor B-cells**	HR 0.97 (CI 0.948 - 0.993), p = 0.011	n.s	HR 0.96 (CI 0.936 - 0.9879), p =0.005	HR 0.970 (CI 0.951 - 0.990), p = 0.0036	n.s.	HR 0.97 (CI 0.95 - 0.99), p = 0.003
**Myeloid progenitors**	HR 1.13 (CI 1.017 - 1.26), p = 0.02	n.s	HR 1.17 (CI 1.023 - 1.328), p= 0 .022	HR 1.21 (CI 1.11 - 1.32), p < 0.001	n.s.	HR 1.48 (CI 1.3-1.76), p < 0.001
**Lymphocytes**	n.s.	n.s.	n.s.	n.s.	n.s.	HR 0.98 (CI 0.9524-0.999), p < 0.05
**Neutrophils**	n.s.	n.s.	n.s.	n.s.	n.s.	n.s.
**Erytroid progenitors**	HR = 0.97 (CI 0.933 - 0.998), p = 0.039	n.s	HR 0.96 (CI 0.931-0.999), p = 0.045	n.s	n.s.	n.s
**pDCs**	n.s.	HR 11.8 (CI 1.407 - 99.73), p = 0.023	n.s.	n.s	n.s.	n.s
**Basophils**	n.s.	n.s	n.s.	n.s	n.s.	n.s
**Eosinophils**	n.s.	n.s.	n.s.	n.s.	n.s.	n.s.

Table S6a: Number of genes mutated in patients with non-del(5q) and del(5q) MDS (HOVON89)

n of mutations:	n of patients	%
**0**	30	22.6
**1**	37	27.8
**2**	33	24.8
**3**	23	17.3
**4**	8	6.0
**5**	2	1.5
**Total:**	133	100

Table S6b: NGS-defined parameters as predictors of HI-E and duration

	HI_E			Duration HI_E		
	**All MDS**	**Del(5q) MDS**	**non-del(5q) MDS**	**All MDS**	**Del(5q) MDS**	**non-del(5q) MDS**
**Mutations**						
**ASXL1**	n.s.	n.s.	n.s.	n.s.	n.s.	n.s.
**CBL**	n.s.	n.s.	n.s.	n.s.	n.s.	n.s.
**NPM1**	n.s.	n.s.	n.s.	n.s.	n.s.	n.s.
**DNMT3A**	n.s.	n.s.	n.s.	n.s.	n.s.	n.s.
**ETV6**	n.s.	n.s.	n.s.	n.s.	n.s.	n.s.
**EZH2**	p = 0.069, 42% vs 0%	n.s.	n.s.	n.s.	n.s.	n.s.
**FLT3**	n.s.	n.s.	n.s.	n.s.	n.s.	n.s.
**IDH1**	n.s.	n.s.	n.s.	n.s.	n.s.	n.s.
**IDH2**	n.s.	n.s.	n.s.	n.s.	n.s.	n.s.
**JAK2**	n.s.	n.s.	n.s.	n.s.	n.s.	n.s.
**KRAS**	n.s.	n.s.	n.s.	n.s.	n.s.	n.s.
**NRAS**	n.s.	n.s.	n.s.	n.s.	n.s.	n.s.
**RUNX1**	n.s.	n.s.	n.s.	n.s.	n.s.	n.s.
**SF3B1**	p = 0.038, 53 % vs 31 %	n.s.	n.s.	n.s.	n.s.	n.s.
**SRSF2**	p = 0.051, 48% vs 11 %	n.s.	n.s.	n.s.	n.s.	n.s.
**TET2**	n.s.	n.s.	n.s.	n.s.	n.s.	n.s.
**TP53**	n.s.	n.s.	n.s.	n.s.	n.s.	n.s.
**U2AF1**	n.s.	n.s.	p = 0.051, 31% vs 75%	p= 0.017, 2.6 % vs 10.1 %	n.s.	p = 0.045, 2.6 % vs 9.2 %
**ZRSR2**	n.s.	n.s.	n.s.	n.s.	n.s.	n.s.
**SF3B1 WT of VAF <20 vs VAF>20**	p = 0.00034, 57% vs 23%	p = 0.012, 100%, 25%	p = 0.061	n.s.	n.s.	n.s.
**Mutations present**	p < 0.0001, 73% vs 34%	n.s.	p = 0.0025, 55% vs 28%	p = 0.04, 83% vs 17.7%	n.s.	p = 0.056, 83% vs 46%
**Number of mutations**	p < 0.0001, 73% vs 34%	n.s.	p = 0.0025, 55% vs 28%	p = 0.04, 83% vs 17.7%	n.s.	p = 0.056, 83% vs 46%

Table S6c: NGS-defined parameters as predictors of OS and PFS

	OS			PFS		
	All MDS	Del(5q) MDS	non-del(5q) MDS	All MDS	Del(5q) MDS	non-del(5q) MDS
**Mutations**						
**ASXL1**	n.s.	n.s.	n.s.	n.s.	n.s.	n.s.
**CBL**	p = 0.087	n.s.	n.s.	n.s.	n.s.	n.s.
**NPM1**	n.s.	n.s.	n.s.	n.s.	n.s.	n.s.
**DNMT3A**	n.s.	p = 0.076	n.s.	n.s.	p =0.0057, 11% vs 0%	n.s.
**ETV6**	n.s.	n.s.	n.s.	n.s.	n.s.	n.s.
**EZH2**	p = 0.018, 36% vs 14%	n.s.	p = 0.016, 37% vs 14%	p < 0.0001, 12% vs 0%	p <0.001 13% vs 0%	p <0.001 13% vs 0%
**FLT3**	n.s.	n.s.	n.s.	n.s.	n.s.	n.s.
**IDH1**	n.s.	n.s.	n.s.	n.s.	n.s.	n.s.
**IDH2**	n.s.	n.s.	n.s.	n.s.	n.s.	n.s.
**JAK2**	n.s.	n.s.	n.s.	n.s.	n.s.	n.s.
**KRAS**	n.s.	n.s.	n.s.	n.s.	n.s.	n.s.
**NRAS**	n.s.	n.s.	n.s.	n.s.	n.s.	n.s.
**RUNX1**	p = 0.01, 34% vs 14%	n.s.	p = 0.0079, 35% vs 14%	p = 0.0035, 10% vs 7%	n.s.	p = 0.003, 11% vs 7%
**SF3B1**	n.s.	n.s.	n.s.	n.s.	n.s.	n.s.
**SRSF2**	n.s.	n.s.	n.s.	n.s.	n.s.	n.s.
**TET2**	n.s.	n.s.	n.s.	n.s.	n.s.	n.s.
**TP53**	n.s.	n.s.	n.s.	n.s.	n.s.	n.s.
**U2AF1**	n.s.	n.s.	n.s.	p = 0.03, 11% vs 0%	n.s.	p = 0.033, 12% vs 0%
**ZRSR2**	n.s.	n.s.	n.s.	n.s.	n.s.	n.s.
**SF3B1 WT or VAF <20 vs VAF>20**	n.s.	n.s.	n.s.	n.s.	n.s.	n.s.
**Mutation present**	p = 0.0041, 60% vs 28%	p = 0.066	p = 0.014, 60% vs 29%	p = 0.021, 30% vs 8%	n.s.	p = 0.0085, 44% vs 8%
**Number of mutations**	p = 0.0083, 60% vs 38% vs 18% vs 35% vs 11%	n.s.	p = 0.028, 60% vs 42% vs 20% vs 32% vs 11%	n.s.	n.s.	p = 0.037

Supplementary figures:

Figure S1: Randomization scheme of HOVON89 arm A vs. arm B (see also prococol supplement 1).

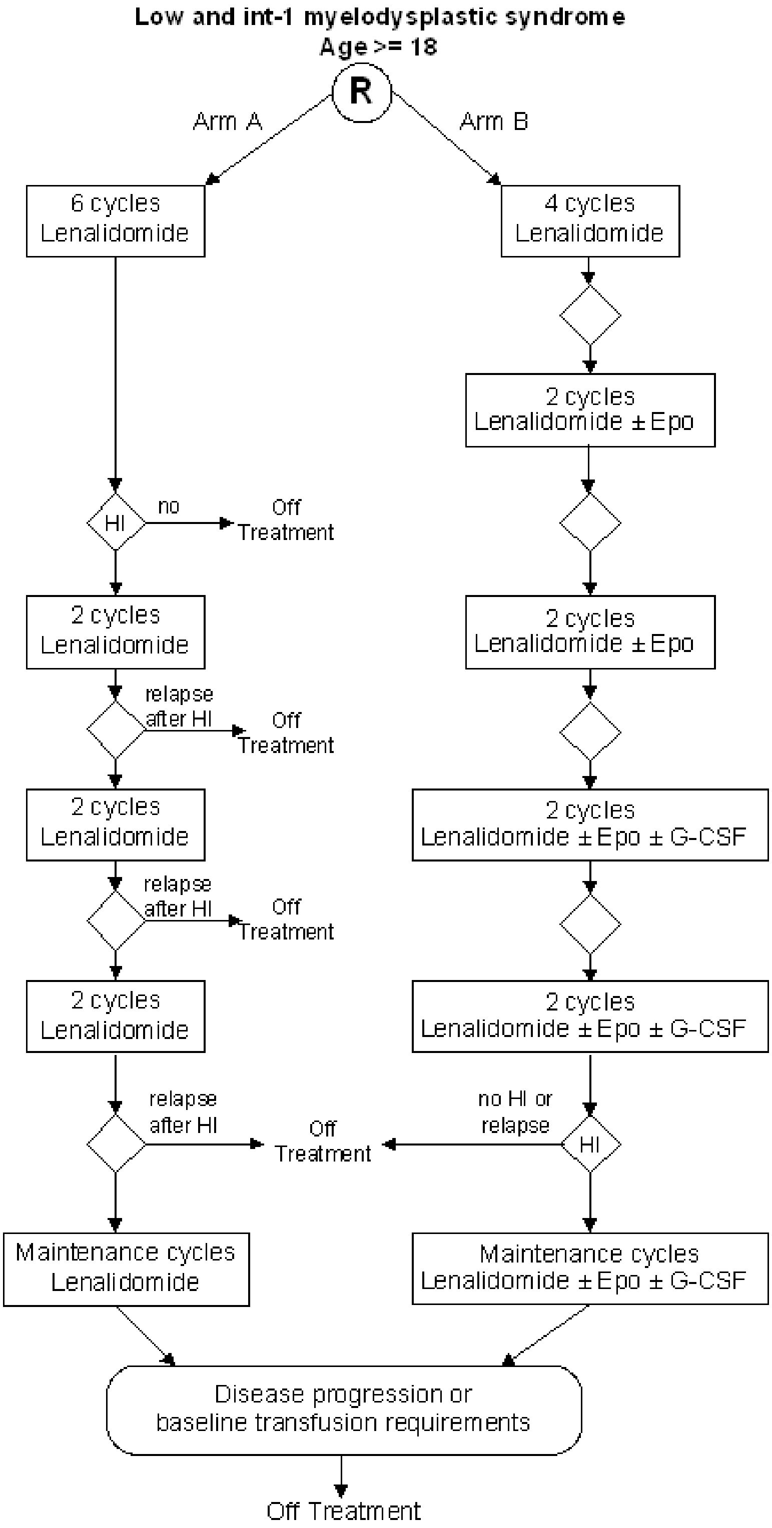

Figures S2: Kaplan-meier estimated leukemic evolution according to arm A and arm B in HOVON89.

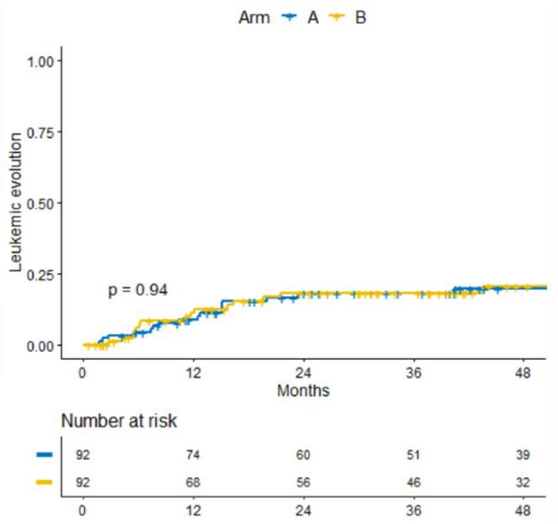

Figures S3a-c: Kaplan-meier estimated overall survival stratified for pretreament with ESA/G-CSF (fig. S3a); Kaplan-meier estimated overall survival and progression free survival according to IPSS risk (fig. 3b-c).

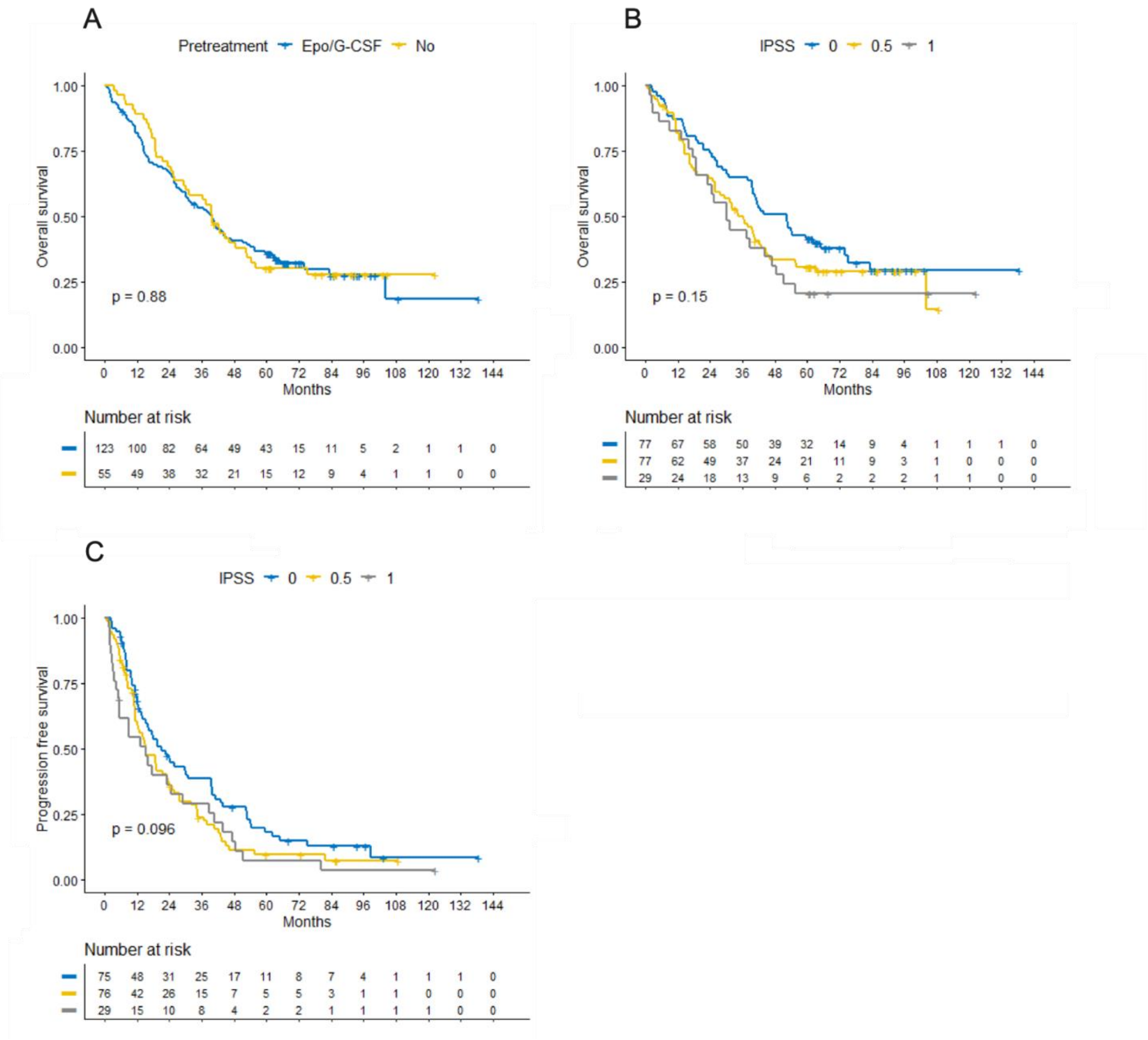

Figures S4a-b: Kaplan-meier estimated overall survival and progression free survival for non-del(5q) and del(5q) MDS in HOVON89.

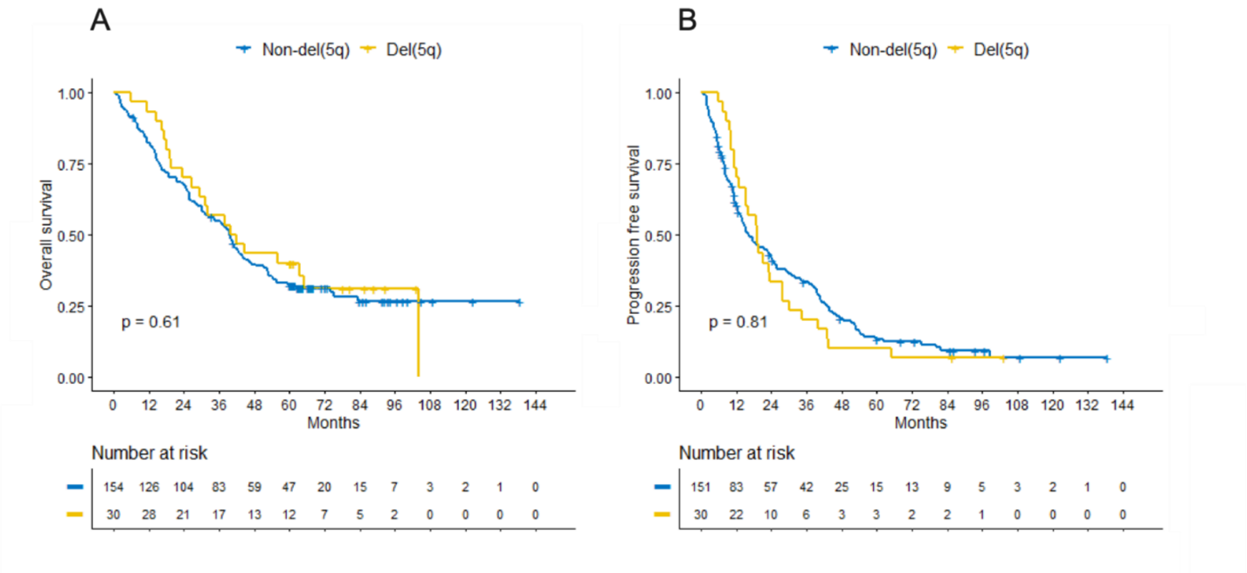

Figures S5a-c: Landmark analysis at 12 months: Kaplan-meier estimated overall survival according to achievement of HI-E for a) all patients within HOVON89; b) for patients with MDS del(5q) and c) for patients with non-del(5q) MDS.

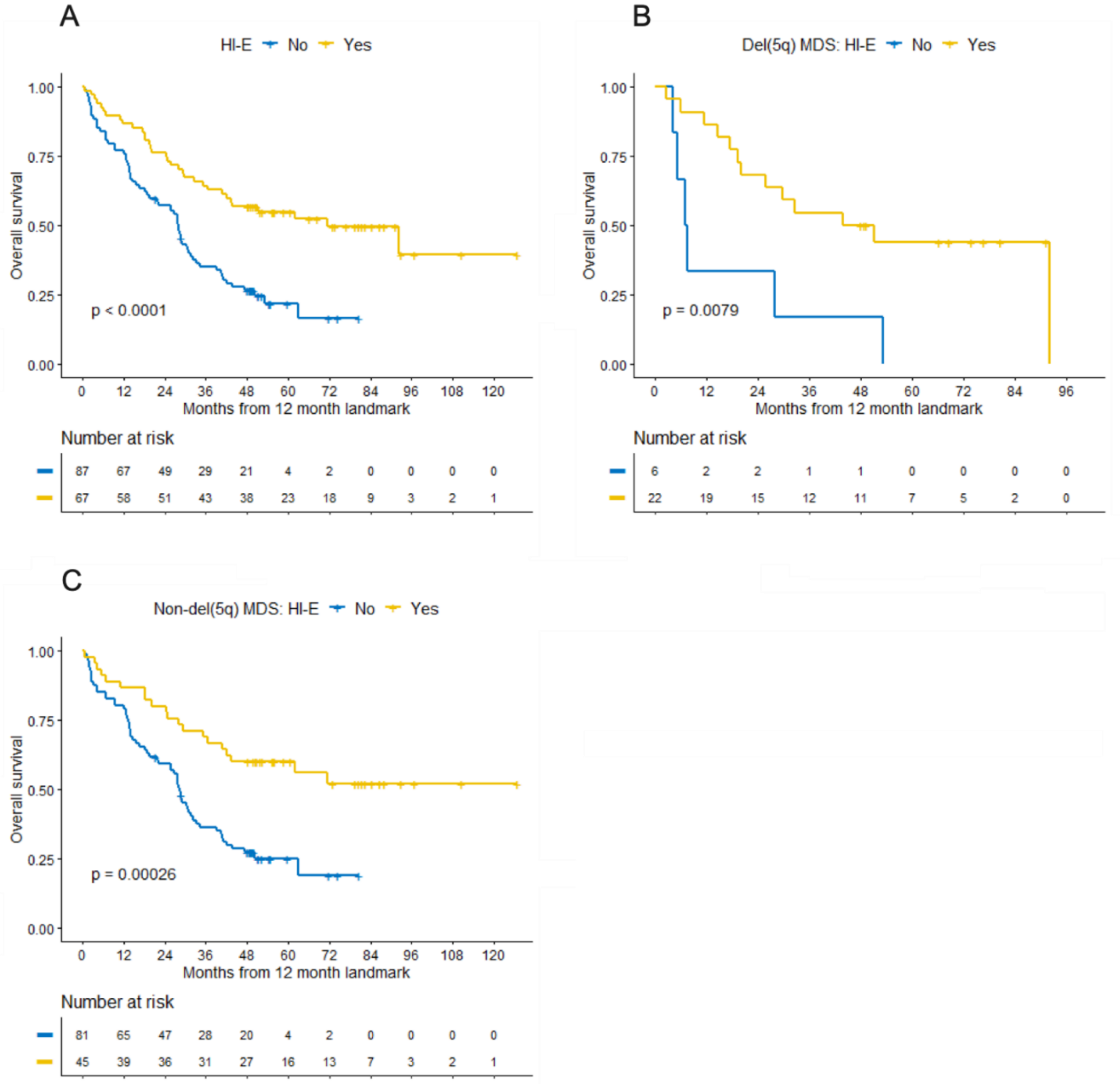

Figure S6: Overview of mutations and co-mutations per patient within HOVON89.

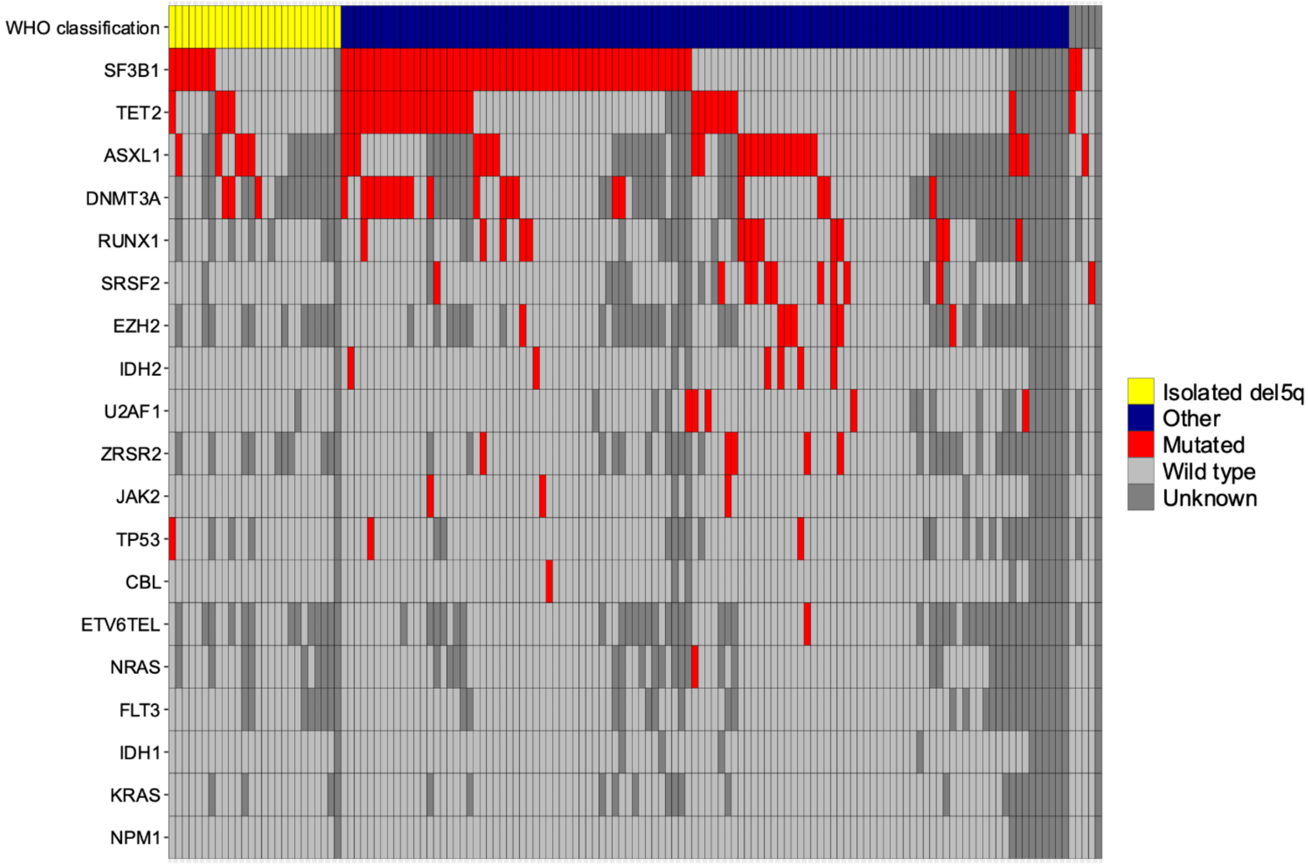

Figures S7a and S7b: Kaplan-meier estimated HI-E by number of mutations in non-del(5q) (a) and del(5q) MDS (b) in HOVON89.

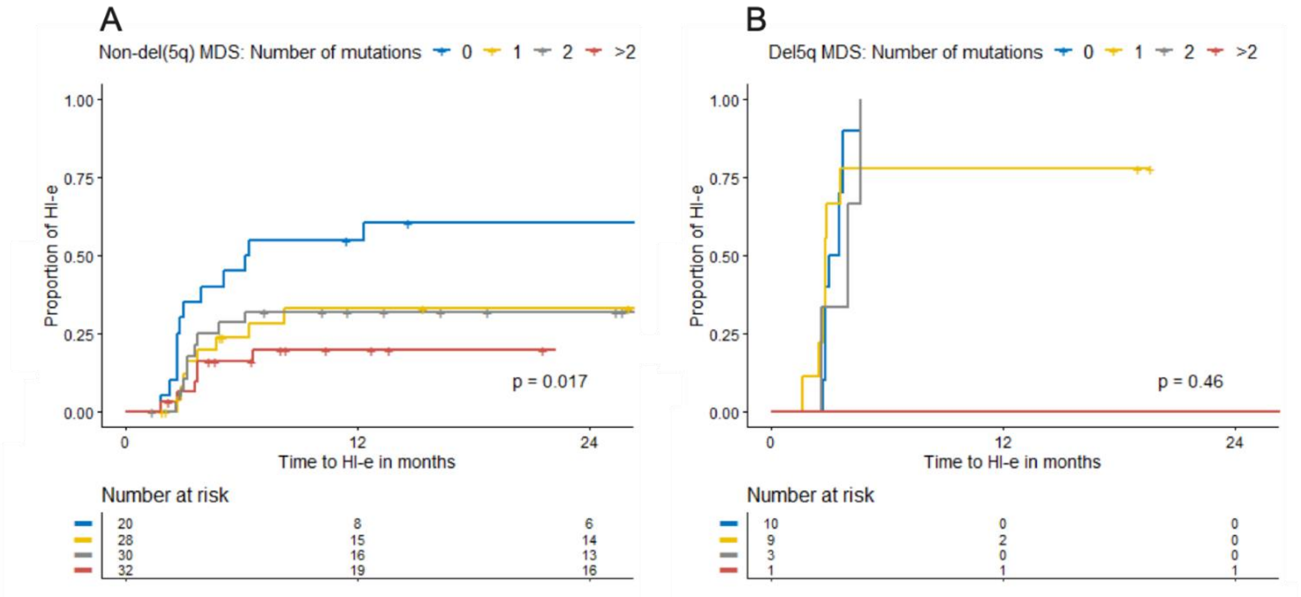

Supplement: Supplementary file 2 — supplementary figures and tables [file 41375_2024_2161_MOESM2_ESM.pdf]
